# A Computational Method for the Binding Mode Prediction of COX-1 and COX-2 Inhibitors: Analyzing the Union of Coxibs, Oxicams, Propionic and Acetic Acids

**DOI:** 10.3390/ph16121688

**Published:** 2023-12-04

**Authors:** Estefany Bello-Vargas, Mario Alberto Leyva-Peralta, Zeferino Gómez-Sandoval, Mario Ordóñez, Rodrigo Said Razo-Hernández

**Affiliations:** 1 Centro de Investigaciones Químicas, Instituto de Investigación en Ciencias Básicas y Aplicadas, Universidad Autónoma del Estado de Morelos, Cuernavaca 62209, Mexico; estefany.bellovar@uaem.edu.mx; 2 Departamento de Ciencias Químico Biológicas y Agropecuarias, Universidad de Sonora, H. Caborca, Sonora 83621, Mexico; mario.leyva@unison.mx; 3 Facultad de Ciencias Químicas, Universidad de Colima, km 9 Carretera Colima-Coquimatlán, Coquimatlán 28400, Mexico; zgomez@ucol.mx; 4 Laboratorio de Quimioinformática y Diseño de Fármacos, Centro de Investigación en Dinámica Celular, Instituto de Investigación en Ciencias Básicas y Aplicadas, Universidad Autónoma del Estado de Morelos, Cuernavaca 62209, Mexico

**Keywords:** anti-inflammatory, cyclooxygenase (COXs), molecular docking, NSAIDs, Celecoxib, Meloxicam, Ibuprofen, Indomethacin

## Abstract

Among the biological targets extensively investigated to improve inflammation and chronic inflammatory conditions, cyclooxygenase enzymes (COXs) occupy a prominent position. The inhibition of these enzymes, essential for mitigating inflammatory processes, is chiefly achieved through Non-Steroidal Anti-Inflammatory Drugs (NSAIDs). In this work, we introduce a novel method—based on computational molecular docking—that could aid in the structure-based design of new compounds or the description of the anti-inflammatory activity of already-tested compounds. For this, we used eight crystal complexes (four COX-1 and COX-2 each), and each pair had a specific NSAID: Celecoxib, Meloxicam, Ibuprofen, and Indomethacin. This selection was based on the ligand selectivity towards COX-1 or COX-2 and their binding mode. An interaction profile of each NSAID was compiled to detect the residues that are key for their binding mode, highlighting the interaction made by the Me group. Furthermore, we rigorously validated our models based on structural accuracy (RMSD < 1) and (R^2^ > 70) using eight NSAIDs and thirteen compounds with IC_50_ values for each enzyme. Therefore, this model can be used for the binding mode prediction of small and structurally rigid compounds that work as COX inhibitors or the prediction of new compounds that are designed by means of a structure-based approach.

## 1. Introduction

Inflammation constitutes a natural protective response in tissues affected by physical trauma, noxious chemicals, or microbial agents. It represents the body’s response to inactivate or eliminate invading pathogens, removing irritants, and laying the foundation for tissue repair [1,2,3,4]. Based on its physiological characteristics, inflammation can be classified as acute or chronic, with chronic inflammation of major significance in the health realm due to its prevalence in chronic pathologies [5,6,7,8].

Concurrently, the role of methyl groups in modulating the biological activity of small molecules is well described in the literature [9]. Numerous research groups have carried out studies on the replacement of hydrogen atoms with methyl groups, consistently demonstrating a favorable impact on the structure–activity relationship of these compounds.

Recent research has revealed how, in SAR studies, there is a 10-fold or greater increase in biological activity in 8% of cases, while a mere 0.4% exhibit a 100-fold or greater increase in biological value. This is related to the placement of a methyl group in a hydrophobic environment that may be suitable; however, orthomethylation in biaryl systems or via branching on an atom attached to a ring favors a conformational change in the biological system. For this reason, the implementation of methyl groups has emerged within the design of bioactive molecules, given the diverse range of advantages it offers [10].

In this context, in recent years, our investigation group has focused on the synthesis of small molecules that contain a methyl group within their structure, which have been shown to exhibit anti-inflammatory activity in vivo models. However, the pharmacological mechanism for this type of molecule has not yet been explored [11].

Although there are various pharmacological mechanisms involved in inflammatory processes [12], the best known and studied is the inhibition of cyclooxygenase enzymes (COXs)—key isoenzymes in the regulation of inflammatory processes—that are essential for the biosynthesis of prostaglandins through the oxidation of arachidonic acid [13]. For this reason, the inhibition of these isoenzymes is one of the topics of study in various research groups with a specific interest in the COX-2 isoform since it has been shown that this is the main participant in inflammatory processes. In addition, there are structural differences such as size, hydrophobicity, and residues within the catalytic site between both isoenzymes [14]. Consequently, the development of new molecules is sought, which present greater selectivity for COX-2.

Given the diversity of available COX crystals in databases and the structural variations in their co-crystallized molecules, choosing suitable crystals for descriptive or predictive computational analysis has become increasingly challenging. Incorrect crystal selection can lead to erroneous molecular designs or an inadequate description of the binding mode of COX inhibitors. Hence, this work introduces a method aimed at streamlining the adequate selection of COX crystals, ultimately bolstering the reliability of the results obtained from molecular docking calculations. This method explores the binding mode of four different NSAIDs: Celecoxib, Meloxicam, Ibuprofen, and Indomethacin. This study demonstrates that it is possible to identify the binding mode of COX inhibitors. Additionally, this method enables the correlation between the interaction energy ($E_{\mathrm{int}}$) obtained via molecular docking calculations with the IC_50_ values obtained from COX inhibition experiments.

## 2. Results and Discussion

### 2.1. Cavities Analysis of COXs

**Figure 1:**
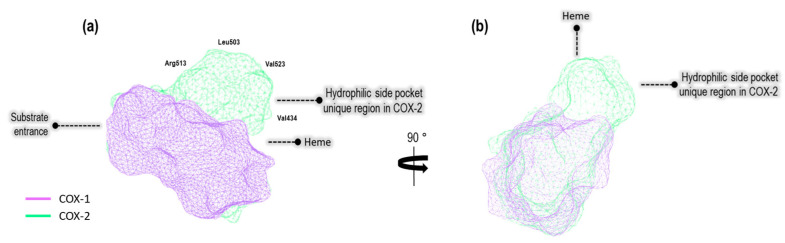
(**a**) The binding site cavity of COX-1 (1EQG) and COX-2 (3LN1), (**b**) turned to the right at 90°.

By conducting a structural analysis using the Protein Pluss server on both COX isoform cavities, we obtained the results depicted in Figure 1.

Based on differences in the structural attributes of these proteins, we identified a hydrophilic pocket unique to COX-2 when compared to COX-1. Additionally, we employed the DoGSiteScorer method, which is designed to detect pockets solely based on the 3D structure of the enzyme and further segment them into sub-pockets [15]. This analysis encompassed the assessment of global properties, including the size, shape, and chemical characteristics of these sub-pockets [16]. The properties under evaluation included the volume (Å^3^), surface area (Å^2^), hydrophobicity depth ratio (Å), and amino acid residues in both, as well as exclusively amino acid residues specific to our model crystals, as illustrated in Table 1.

The properties reveal that the COX-2 (4PH9, 3LN1, and 4M11) cavity exhibits a large volume (Å^3^) and greater surface area (Å^2^) compared to the COX-1 (1EQG, 3LN1, and 4O1Z) cavity. Also, the COX-2 catalytic site is deeper, according to Table 1 (except for the 2OYU crystal, Indomethacin). However, in the system of Indomethacin (2OYU and 4COX), the relationship with respect to the values mentioned above is inverse due to the structural difference in the co-crystalized ligands. In the crystal 2OYU, the ligand is Indomethacin-(*S*)-alpha-ethyl-ethanolamide (Indomethacin alpha) with a higher volume than Indomethacin, which is a ligand present in the crystal 4COX. With respect to the aminoacid residues, the following Tyr355, Arg120, Ser530, and Tyr385 are retained in all systems. On the other hand, the residues Ile523, Ile434, Phe503, and His513 are present only in the COX-1 cavity, and Leu503, Val384, Val523, and Arg513 are present only in the COX-2 cavity.

In addition, bioinformatic analysis was used to compare the sequences of all COX-1 and COX-2 crystals (see Figure S1, COX-1 and Figure S2, COX-2). For COX-1, three different organisms were analyzed: *Human*, *Mus musculus*, and *Ovis aries*. The focus of this analysis was to identify which aa is conserved in the COX-1 binding site (see Table 1, columns 6 and 7). As a result, only a difference in the *Human* sequence was found (His513 was replaced by Gln); see Figure S1. On the other hand, when analyzing COX2 sequences, we found a complete similarity. Furthermore, we continued with the construction of our computational model since there is no significant variation in the selected crystals.

### 2.2. Collection of Information for the Date (Active Compounds)

**Figure 2:**
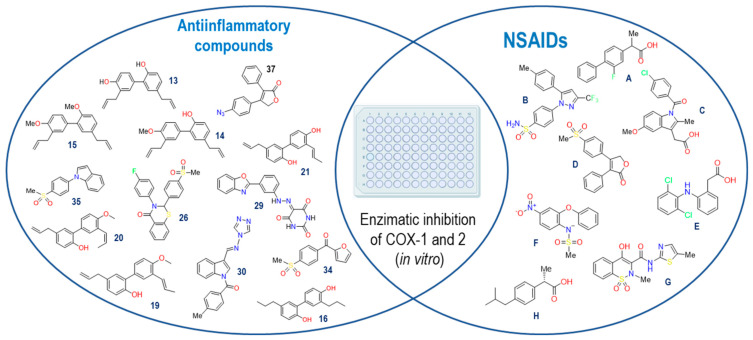
NSAIDs and anti-inflammatory compounds employed in the computational model. Flurbiprofen (**A**), Celecoxib (**B**), Indomethacin (**C**), Rofecoxib (**D**), Diclofenac (**E**), Nimesulide (**F**), Meloxicam (**G**) and Ibuprofen (**H**).

We proceeded with the search for IC_50_ values reported for NSAIDs present in our crystals in the ChEMBL database [17] for both COX isoforms. Additionally, we included the following NSAIDs: Flurbiprofen [18], Diclofenac [19], Rofecoxib [20], and Nimesulide [21]. These compounds served as participants in the structural and energetic validation of our model, leveraging our knowledge of their binding modes and their selectivity towards COX enzymes. Furthermore, we identified fifty compounds with documented anti-inflammatory activity, which were assessed using in vitro models from the literature. However, out of these compounds, only thirteen were selected since they had both IC_50_ values on COXs, like the NSAIDs selected (Figure 2).

The thirteen active compounds we sought had a structural diversity that presented a challenge in our model, and thus, evaluating this variable, even if we could find a correlation between the data predicted by the model with the reported biological activity values, was unfeasible. The criteria used for the selection of these compounds were to maintain at least two aromatic rings within the structures and a molecular weight of fewer than 500 KDa.

### 2.3. Analysis of the Crystal Selected for the Predictive Model

**Figure 3:**
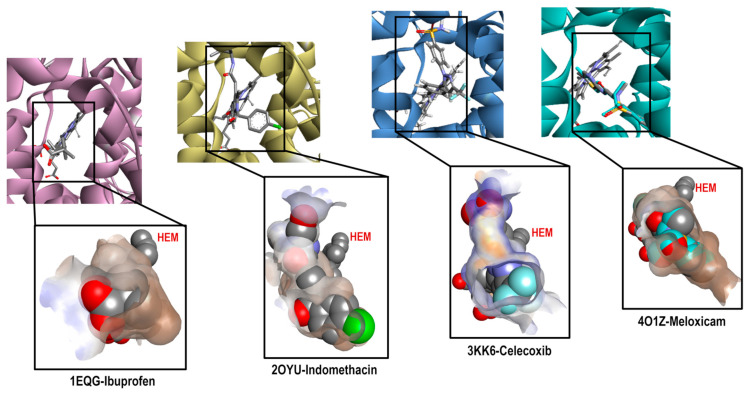
Comparative cavities in various COX-1 crystals with diverse ligands.

Continuing with the elaboration of our model, we previously selected the crystals, and structures of the molecules that would form part of our structural and energetic validation. Therefore, the next step was the preparation and analysis of our selected crystals for COX-1. Figure 3 shows the binding mode of the ligands as follows: Ibuprofen, Indomethacin, Celecoxib, and Meloxicam in the enzyme binding site. We also analyzed the ligand size, shape and hydrophobic characteristics related to the cavity where these ligands were spatially positioned.

By analyzing these crystals (see Figure S3), we found important differences in each system, highlighting that they bound in a different way and at a location of the cavity where arachidonic acid (the endogenous ligand) also binds. For Ibuprofen (1EQG), we observed the proper accommodation of size over the COX-1 cavity, where the aromatic ring was in front of the HEM group and interacted with the following amino acid residues: Ile523, Val349, and Ala527 (Pi-alkyl and carboxylic acid on the back side in a more hydrophilic environment interacted with the following amino acid residues: Arg120 and Tyr355 (Conventional Hydrogen Bond). In the case of the methyl substituent in the alpha position to the carbonyl group, it interacted with the residues Val349, Val116, Leu531, and Leu359 (Alkyl). Finally, the heme-oriented isobutyl group showed interactions with the following amino acid residues: Leu352, Gly256, Phe518, Trp387, Met522, Tyr385, Ser530, and Phe381 (Van der Waals).

Indomethacin (2OYU) was bound in a different mode than the one expected for acetic acid derivatives due to the substituent attached to it (alpha-ethyl-ethanolamide group), modifying the orientation of the methoxy group attached to the indole ring to the HEM group, which is a position normally occupied by the ρ-chlorobenzene ring. The interactions observed for Indomethacin alfa were as follows: the ρ-chlorobenzene ring with residue amino acids Tyr355 (Pi-Pi Stacked), Met113 (Pi-Sulfur), Val116, Leu359, Val349 and Leu531 (Alkyl); the amide cyclic with Arg120 (Conventional Hydrogen Bond); the ring of indole with Val349 and Ala527 (Pi-Alkyl); and the MeO group with Trp387 (Pi-Alkyl) and Leu384 (Alkyl). Lastly, the ethyl-ethanolamide group interacted with Ile517 (Alkyl), His90 (Pi-Alkyl), and Phe518 (Pi-Alkyl) (see Figure S4).

For Celecoxib (3KK6), a C-type accommodation was displayed, occupying a larger space than Ibuprofen with the toluene fragment looking towards the HEM; this interacted with Trp387, Tyr385 and Ala527 (Pi-Alkyl), Leu352 (Pi-Sigma); for the pyrazole ring with Ala527 and Val349; the CF_3_ group interacted with Val116, Leu359 and Val349 (Alkyl); and interactions with a hydrophilic pocket were shown for His90 (Pi-Sulfur and Carbon Hydrogen Bond), Ser516 (Carbon Hydrogen Bond), Leu352, and Gln192 (Conventional Hydrogen Bond) by its sulfonamide group (see Figure S5).

Finally, Meloxicam (4O1Z) displayed a different binding mode from those mentioned above, revealing a new hydrophobic pocket in the binding site where the benzothiazine ring—characteristic of the enolic acid derivatives (oxicams)—was harbored. This ring interacted with the following next residue amino acids: Leu531 (Pi-Sigma), Ile345 and Val349 (Pi-Alkyl), and Ser530 (Conventional Hydrogen Bond); the Thiazole ring interacted with Ile523, Ala527, Phe518, Leu384, Met522, and Trp (Pi-Alkyl), Gly526 (Amide-Pi Stacked), and Leu352 (Pi-Sigma) (see Figure S6). This finding is of great importance for the description of the binding mode of new inhibitors because, generally, this binding mode is not considered.

This analysis shows the importance of the structural and physicochemical properties of the ligand in its interaction with the enzyme. Although the chemical environment at the binding site was the same (Ibuprofen, Indomethacin, and Celecoxib), the differences in size, structure, and hydrophobicity between these molecules generated a different binding mode. In addition, in the case of Meloxicam, a greater difference was achieved, revealing a different pocket that needs to be considered for molecular docking prediction. Thus, this reaffirms the selection of the four crystals with their corresponding ligands for the construction of our predictive model.

**Figure 4:**
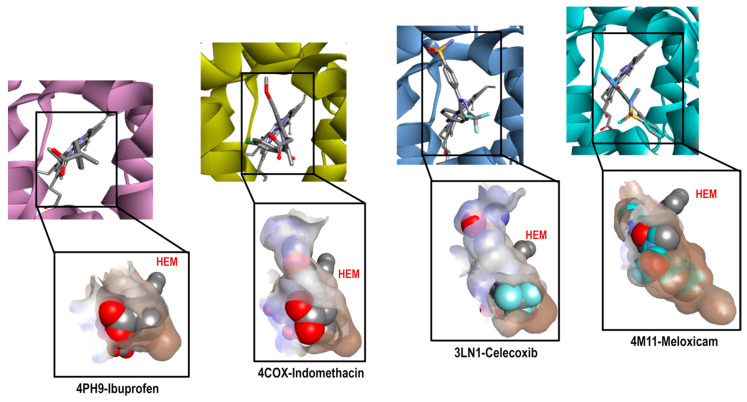
Comparative cavities in various COX-2 crystals with diverse ligands.

The same analysis was performed with COX-2 crystals. Figure 4 shows the binding mode of the ligands as follows: Ibuprofen (4PH9), Indomethacin (4COX), Celecoxib (3LN1), and Meloxicam (4M11) on the COX-2 enzyme. The ligand analysis was performed based on their size, molecular shape, hydrophobic characteristics, and interaction residues.

Figure 4 shows how the binding modes of Ibuprofen, Celecoxib, and Meloxicam maintain great similarity when compared to COX-1. The interactions with Ibuprofen in crystal 4PH9 are described next. Carboxylic acid interacted only with the following amino acid residues: Arg121 and Tyr356 (Conventional Hydrogen Bond) on the back side in an environment that was more hydrophilic, as well as COX-1. For the methyl substituent in the alpha position to the carbonyl group, it interacted with the residues Leu360, Leu532, Val117, Tyr356 and Val350 (Alkyl); the ρ- isobutylbenzene group showed interactions with Ala528 and Val350 (Pi-Sigma), Ser531, Tyr386 and Val524 (Van der Waals) (see Figure S7).

However, the case of the 4COX crystal is totally different since Indomethacin has no structural modification like the one in the 2OYU crystal. In 4COX, the carboxylic acid group of Indomethacin is oriented to the segment with higher hydrophilicity interacting with Arg120 (Attractive Charge) and Tyr355 (Conventional Hydrogen Bond); the ring of indole interacted with Val349, Val523, and Ala527 (Pi-Sigma), Leu531, Val349, Val523, Leu352, Ala527 (Pi-Alkyl), His90 (Pi-Alkyl), Leu352, Ser353 (Van der Waals). Meanwhile, the ρ-chlorobenzene ring interacted with Tyr385 (Pi-Pi-shaped), Ala527, Met522, Trp387, and Leu384 (Pi-alkyl and Alkyl) in front of the HEM group (see Figure S8).

In this analysis, the interactions obtained for celecoxib in crystal 3LN1 were as follows: toluene groups interacted with Tyr371, Trp373, Leu370 (Pi-Alkyl and Alkyl), Gly512 (Amide-Pi Stacked), and Ala513 (Pi-Sigma); the CF_3_-Pyrazole group interacted with Ala513, Val335 (Pi-Sigma), Arg106 (Conventional Hydrogen Bond), Leu517, and Val335 (Alkyl). For the third aromatic ring in the structure (Benzenesuldidfonamide), the observed interactions were as follows: Val509, Ser339 (Pi-Sigma), Ser339, Gln178, Leu338, Arg499 (Conventional Hydrogen Bond) (see Figure S9).

Finally, the interactions for Meloxicam in 4M11 could be divided into two regions; in the first one, we found the thiazine ring, which interacted with Ser530 (Conventional Hydrogen Bond), Leu531, Ile345 (Pi-Alkyl), and Met535 (Pi-Sulfur). In the second, we found thiazole ring interactions with Val523, Met522, Ala527, Phe518 (Pi-Alkyl), Trp387 (Pi-Sulfur), Gly(526), and Leu (Pi-Sigma) (see Figure S10).

After obtaining the complete analysis of the binding modes of these NSAIDs, we decided to continue with the next step of our method: computational molecular docking.

### 2.4. Structural Validation of the Docking Model

**Figure 5:**
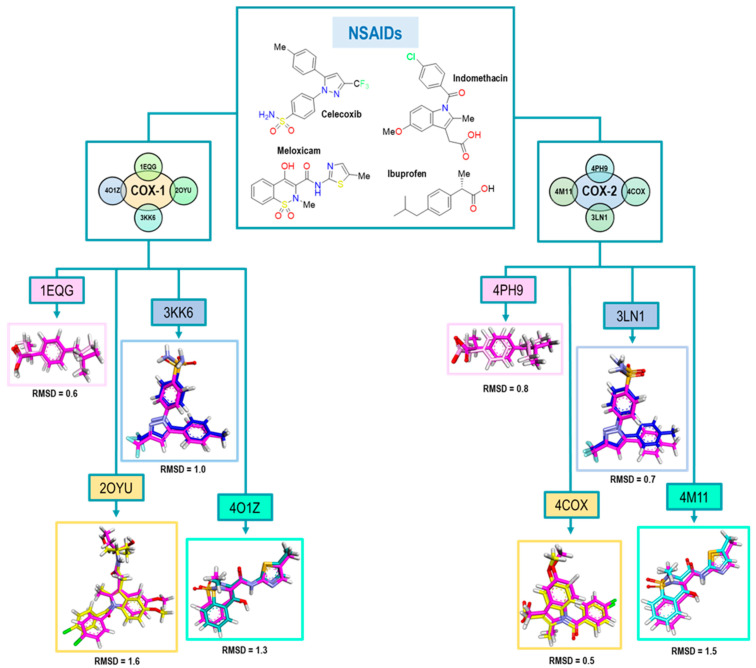
RMSD values obtained for the structural validation of the computational model with NSAIDs where the molecule is the original pose in all cases and is the color magenta. Colors for each pose represent those obtained in the molecules after the experiment as follows: Ibuprofen (pink), Indomethacin (yellow), Celecoxib (blue) and Meloxicam (cyan).

The next step in our method consisted of the ability to predict the binding mode of new compounds, considering the four binding modes previously described. Therefore, the validation of computational molecular docking was fundamental to evaluate the predictive ability of our method. To accomplish this, for each system, the original conformation of the ligand in the crystal was compared to the one obtained from the molecular docking. For the evaluation of conformational reproducibility, we employed the root mean square deviation (RMSD) value, as shown in Figure 5.

The results obtained for Ibuprofen, Indomethacin, Celecoxib, and Meloxicam are in a range of an RMSD lower than 1.6, where Ibuprofen exhibited the best value; see Figure 5. The highest RMSD value was obtained for the 2OYU crystal because of its Indomethacin derivative co-crystallized. Nevertheless, its RMSD value met the acceptability criteria. For the case of Celecoxib, we obtained a value of 1.0; both conformations are quite similar, although, in the pose from the docking experiment, the sulfonamide group was found with a small 90° twist to the right.

Finally, in the 4O1Z crystal with Meloxicam as the ligand, we noticed that two different conformations for the ligand were displayed. The first one showed the parallel orientation of the sulfur atom of the thiazole ring with the carbonyl of the amide. For the second orientation, the oxygen atom of the thiazole ring was parallel (see Figure S11). However, we decided to take the first conformation as the biologically active one, according to Meanweel et al. [20].

For COX-2 crystals, we achieved better RMSD values, especially in the case of Indomethacin and Celecoxib. We associated this with the structure of Indomethacin, which has nonstructural modifications like 2OYU. Therefore, based on these RMSD values (see Figure 5), we concluded that our method had an adequate acceptability criterion for the structural results (conformations) obtained from the docking.

#### Validation of the $E_{int}$ Values for Our Computational Model

**Figure 6:**
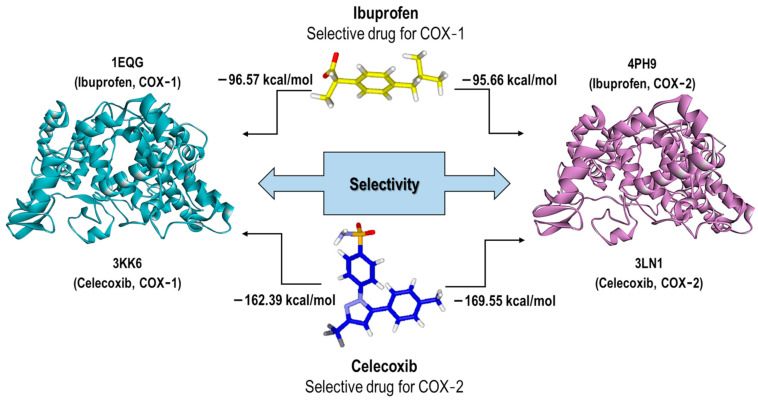
The scheme of our method validation in relation to the selectivity and $E_{int}$. As an example, the Ibuprofen and Celecoxib validation process is depicted.

Regardless of the structural validation, each docking result (pose) was accompanied by an energetic value related to its stability, which could be correlated to experimental inhibition values (IC_50_). Therefore, a validation of our method related to the interaction energy values ($E_{int}$) obtained from the docking calculations was necessary. Therefore, we performed this validation for each of the co-crystallized NSAIDs and another four NSAIDs as follows: Flurbiprofen, Nimesulide, Diclofenac, and Rofecoxib. It is worth mentioning that for all these NSAIDs, an IC_50_ value for each COX isoform was obtained from the literature. This knowledge allowed us to evaluate the prediction of our method in relation to the selectivity of each NSAID for both isoforms. Figure 6 shows the analysis of two NSAIDs (non-selective and selective type) on both COX isoforms and the energy values obtained for each of them.

The $E_{int}$ values for Ibuprofen (non-selective NSAID) were −96.57 Kcal/mol and −95.66 Kcal/mol in COX-1 (1EQG) and COX-2 (4PH9), respectively, indicating a lower selectivity towards COX-1. This value correlates with the selectivity reported in the literature. In addition, another factor that we included as critical was the binding mode, which is addressed later. In the case of Celecoxib, which is a selective NSAID towards COX-2, its energetic values were −169.55 Kcal/mol and −162.39 Kcal/mol for COX-2 and COX-1, respectively. This correlation was not obtained for the rest of the systems (COX crystals). Therefore, we undertook a more in-depth visual inspection considering the energy values with the binding mode.

**Figure 7:**
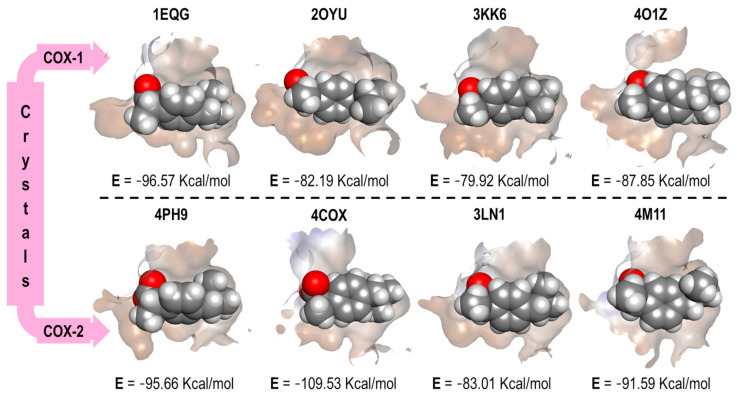
Ibuprofen binding mode and interaction energy values over all COX-1 and COX-2 crystals.

In Figure 7, the four binding modes of Ibuprofen in the different crystals (four for COX-1 and four for COX-2) and the energy values obtained from the docking experiments are shown.

In Figure 7, fewer differences can be distinguished in the binding mode of Ibuprofen in 1EQG and 4PH9 crystals, which acted as our reference for the interaction of Ibuprofen in COX-1 and COX-2. The reproducibility of the binding mode, based on the orientations of its molecular fragments (carboxylic acid and propyl), was evaluated. However, in the other three crystal pairs (Indomethacin, Celecoxib, and Meloxicam), the obtained conformations were considerably different, especially with a disproportional benzene twist and a slight change in the position of the carboxylic acid in the structure, which affected the energy values (Kcal/mol). Therefore, for these three cases, we did not find an energy and selectivity correlation for the ibuprofen structure.

**Figure 8:**
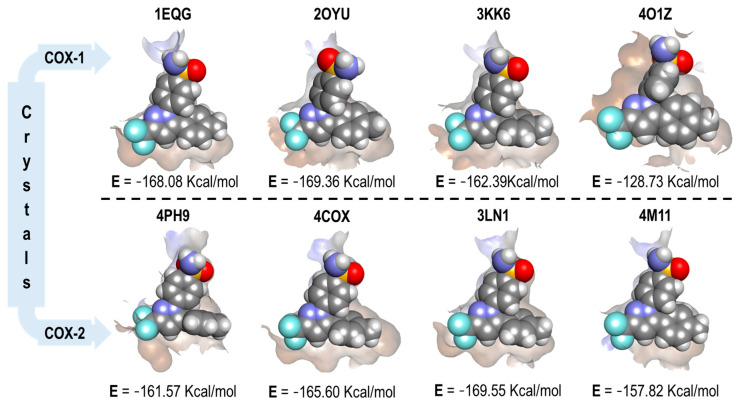
Celecoxib binding mode and interaction energy values over all COX-1 and COX-2 crystals.

Since the co-crystallized structures differ for COX-1 and COX-2, we omitted the comparison described above for Indomethacin. Therefore, we could not perform the same analysis. Figure 8 displays the four binding modes of Celecoxib in all the crystals, along with their corresponding energy values.

From Figure 8, we can visualize the cavity size of the four crystals affected by Celecoxib binding. In the four crystals for COX-1 (top row), we found a similar spatial orientation, except for crystal 2OYU (Indomethacin), where the oxygen of the sulfonamide group was in the opposite position with respect to the reference. After analyzing the binding modes and finding some analogy between them, we evaluated the correlation of the energy with respect to the selectivity of Celecoxib for COX-2. This was achieved in only two pairs of crystals as follows: 3KK6(COX-1) with 3LN1 (COX-2) and 4O1Z (COX-1) with 4M11 (COX-2).

**Figure 9:**
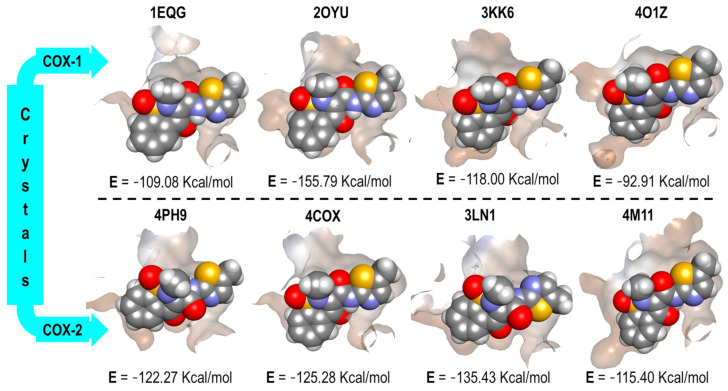
Meloxicam binding mode and interaction energy values over all COX-1 and COX-2 crystals.

Finally, the structural and energetic analysis of Meloxicam was based on its reference crystals 4O1Z (COX-1) and 4M11 (COX-2). In Figure 9, the four binding modes of Meloxicam in the COX crystals are displayed. Additionally, the energy values of each complex and its cavity shape are depicted.

In Figure 9, the different binding modes of Meloxicam over all the COX crystals are depicted. It can be shown that the adequate correlation between the pose and the energy values was obtained only in the 4O1Z and 4M11 crystals pair, which were co-crystalized with Meloxicam. In the other crystal pairs, features like size (1EQG and 4PH9) or the lack of a hydrophobic binding region that the oxicams opened (3KK6 and 3LN1) prevented the correct correlation between the docking results (pose and energy values).

This analysis, together with the visual inspection of these experiments, allowed us to confirm that our enzyme (COX-1 or COX-2) had a conformation and cavity size already adapted to the structural needs of the co-crystallized ligand, which is not necessarily equivalent to all the compounds.

Once the structural and energy data of the reference NSAIDs were analyzed, we decided to carry out this double validation incorporating four more NSAIDs in the study. Table 2 shows the energy values obtained for each NSAID in the eight COXs crystal structures and the energy difference values (ΔE), which represent the difference between the ${\bar{E}}_{COX-1}$ obtained in the COX-1 crystals minus the ${\bar{E}}_{COX-2}$, obtained in the COX-2 crystals ($\Delta E={\bar{E}}_{COX-1}-{\bar{E}}_{COX-2}$), correlating with selectivity towards one of the COX isoforms. Here, ${\bar{E}}_{COX-1}$ stans for the average of all the $E_{int}$ in COX-1 and ${\bar{E}}_{COX-2}$ stands for the average of all the $E_{int}$ in COX-2.

**Figure 10:**
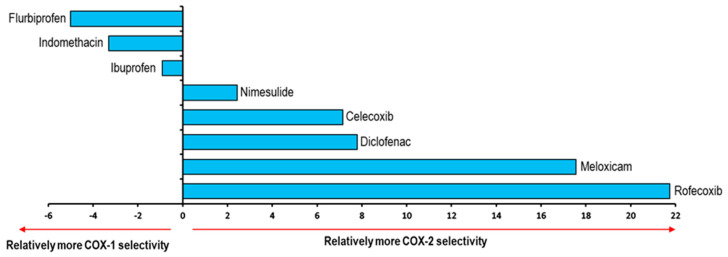
Graphical representation of ΔE values and their correlation to the relative COX-1 and COX-2 selectivity of NSAIDs. Positive values imply COX-2 selectivity and negative values imply COX-1 selectivity.

Negative values of ΔE denote the affinity of the NSAID towards COX-1. On the other hand, positive values of ΔE are related to an affinity towards COX-2, see Figure 10.

These results were compared with data reported in the literature for the selectivity of NSAIDs (Table S1). Flurbiprofen appears as the NSAID with the highest selectivity to the COX-1 isoform, surpassing Indomethacin and Ibuprofen. Moreover, Nimesulide has the lowest selectivity to COX-2. Likewise, Celecoxib with 7.15 and Diclofenac with 7.79 show a great selectivity to this isoform. Finally, Meloxicam and Rofecoxib were those with the most positive values, representing the two NSAIDs with the highest selectivity towards COX-2 according to our model. 2.4.2. This formed the validation of the applicability of our computational method.

### 2.5. Validation of the Applicability Domain of Our Computational Method

- The Ibuprofen case (1EQG and 4PH9)

With the success obtained in the double validation of our model, we decided to evaluate its predictive power in a different system of molecules without a known binding mode. We performed this by adding new molecules with experimental IC_50_ values reported (on both COXs isoforms) in the literature, and we treated them as the NSAIDs previously studied. This was necessary since, based on the $E_{int}$ energy correlation with IC_50_ values, we proposed one of the four binding modes already studied. Therefore, we used 21 molecules for this type of system, performing the molecular docking experiment for each molecule with each of the crystals (8 PDBs) of our model.

**Figure 11:**
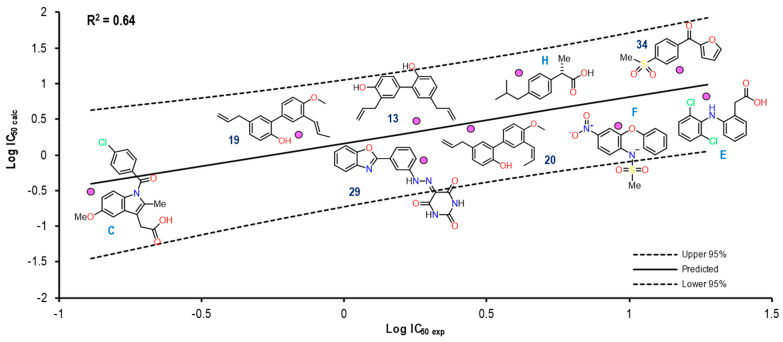
Graphical representation of Log IC_50 calc_ vs Log IC_50 exp_. The 2D structures of molecules that fit in the COX-1 crystal (1EQG) model. Dotted lines show the confidence interval based on the standard deviation.

Figure 11 shows the graph with the values of Log IC_50 exp_ (X-axis) and Log IC_50 calc_ (Y-axis), as well as R^2^ = 0.64. The molecules shown in the graph are those in which it was possible to correlate the $E_{\mathrm{int}}$ with their experimental IC_50_ by means of the IC_50_ obtained from the mathematical model that correlated with the $E_{int}$ obtained from the docking in the 1EQG crystal (see Figure S12).

For the 1EQG crystal, a reproducibility of nine molecules out of 21 was achieved, observing mostly structures with two aromatic rings and only two with a considerable difference in size, which were Indomethacin and compound **29**. However, compound **20** was observed with a position closer to the prediction line in comparison with Ibuprofen, which presented a greater distance from it.

The $E_{int}$, experimental and calculated Log IC_50_ and Δerror (Log IC_50 calc_–Log IC_50 exp_) values are shown in Table S2.

**Figure 12:**
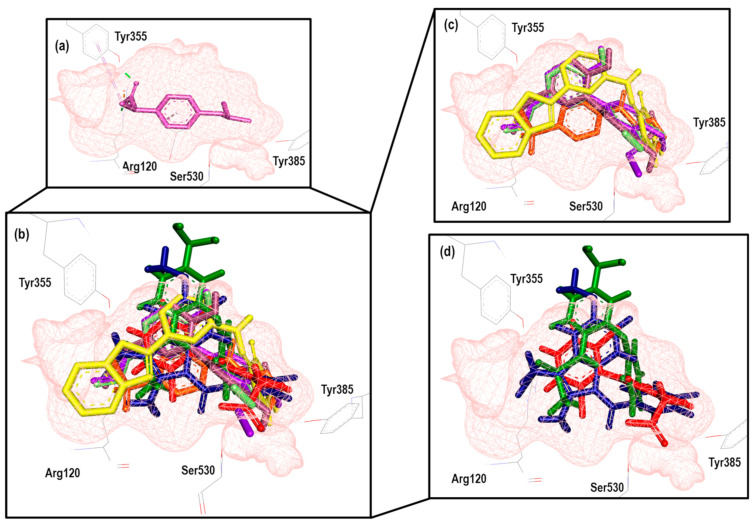
Poses obtained from the docking calculation over the 1EQG crystal. (**a**) Ibuprofen is colored pink. (**b**) The poses of all the ligands that fit the 1EQG model; ligands are colored in the following form: 19 (purple), 20 (aquamarine), 34 (orange), 13 (lilac), Indomethacin (dark blue), Nimesulide (green), 29 (yellow) and Diclofenac (red). (**c**) Ligands 13, 19, 20, 29 and 34 as binding modes. (**d**) The Indomethacin, Nimesulide and Diclofenac binding mode.

To further analyze the relationship between the values obtained from Figure 11 and Table S2, we examined the binding of the nine ligands in the cavity of the 1EQG crystal for COX-1. In Figure 12, all the ligands shown in Figure 11 are displayed in the cavity of COX-1.

We detected a close relationship between the size and shape of the cavity with the ligands that were docked into it. COX-1 of the 1EQG crystal had a cavity volume of 204.80 Å^3^ because of the small size of Ibuprofen. Thus, this limited the size and shape of the binding site and influenced the type of molecules that were docked. Therefore, those that presented greater structural differences in relation to Ibuprofen, such as coxibs or oxicams, were rejected and unable to bind in such a cavity. For the case of Indomethacin and Nimesulide, the nitro and the ρ-MeO groups, respectively, were outside of the cavity.

**Figure 13:**
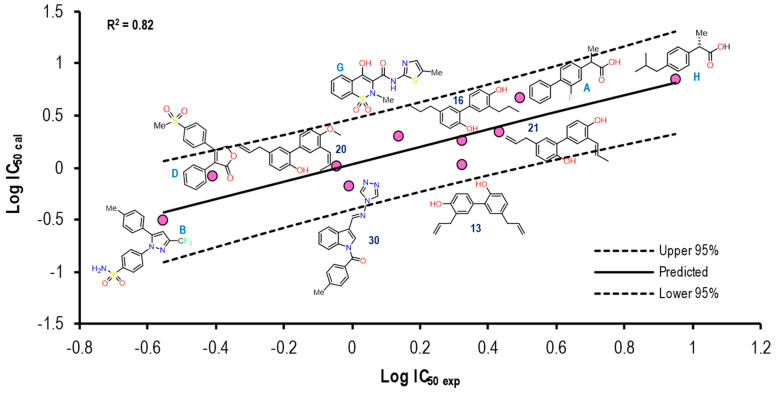
Graphical representation of Log IC_50 calc_ vs Log IC_50 exp_. The 2D structures of molecules that fit in the COX-2 crystal (4PH9) model. Dotted lines show the confidence interval based on the standard deviation.

Figure 13 shows the graph with the values of Log IC_50 exp_ (X-axis) and Log IC_50 calc_ (Y-axis), as well as R^2^ = 0.82. The molecules shown in the graph are those in which it was possible to correlate the $E_{int}$ with their experimental IC_50_ by means of the IC_50_ obtained from the mathematical model correlating $E_{int}$ obtained from the docking in the 4PH9 crystal (see Figure S13).

For the 4PH9 crystal, a reproducibility of ten molecules out of **21** was achieved, which was observed mostly on a structure greater than the reference ligand: Celecoxib, Rofecoxib, Meloxicam, as well as compound **30**. Compounds **16** and **20** showed a similitude to Ibuprofen itself for the predictive line. However, Flurbiprofen did not present the same tendency as Ibuprofen. For the rest of the molecules, we did not observe a considerable distance.

The $E_{int}$, experimental and calculated Log IC_50_ and Δerror (Log IC_50 calc_–Log IC_50 exp_) values are shown in Table S3.

To further analyze the relationship between the values obtained from Figure 13 and Table S3, we examined the binding of the nine ligands in the cavity of the 1EQG crystal for COX-1.

**Figure 14:**
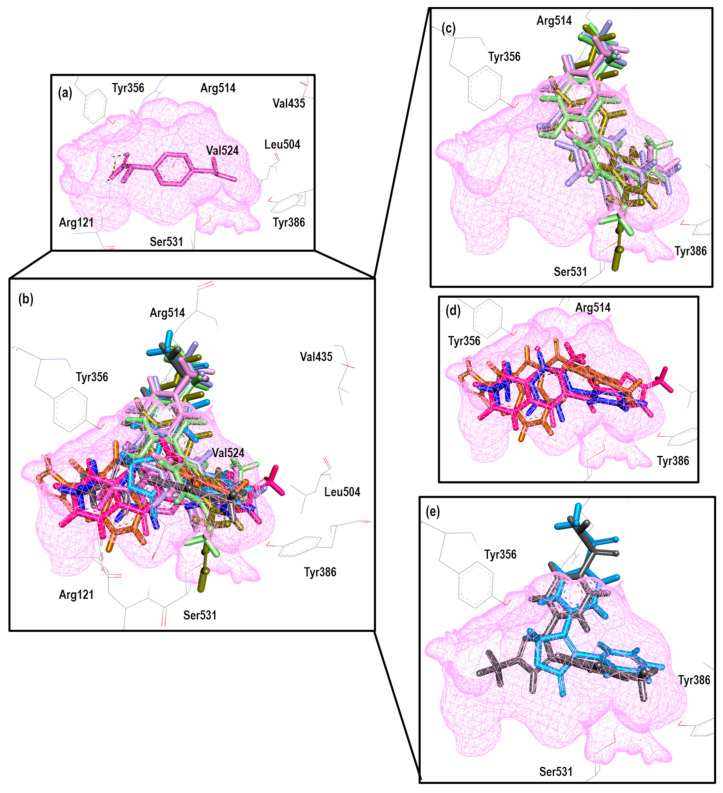
Poses obtained from the docking calculation over 4PH9 crystal (**a**) Ibuprofen is the color pink. (**b**) Poses of all ligands that fit the 4PH9 model; ligands are colored in the following form: 13 (lilac stick), 16 (blue gray), 20 (aquamarine), Flurbiprofen (blue dark), 21 (olive green), Rofecoxib (blue light), Celecoxib (grey), Meloxicam (magenta) and 30 (brown light). (**c**) Ligands 13, 16, 20 and 21 binding modes. (**d**) Meloxicam, Flurbiprofen and 30 binding modes. (**e**) Celecoxib and Rofecoxib binding modes.

Figure 14 shows the binding mode of the 10 ligands in the system of the Ibuprofen crystal (4PH9) of COX-2 (cavity), as well as the characteristic residues for COX-2.

We detected a close relationship between the size and shape of the cavity with the ligands that were docked into it. COX-1 of the 4PH9 crystal had a cavity volume of 228.86 Å^3^. However, even with the small size of Ibuprofen, the volume of the cavity in this crystal was larger because it belonged to COX-2, and there was a difference between the amino acids that made up the binding site. Therefore, they had greater access to the larger molecules of the binding site, such as Celecoxib and Rofecoxib, with similarity in the binding mode. Also, Meloxicam, **30**, and Flurbiprofen were able to fit properly, and for compounds **13**, **16**, **20**, and **21**, we observed a binding mode that was different in comparison to NSAIDs.

**Figure 15:**
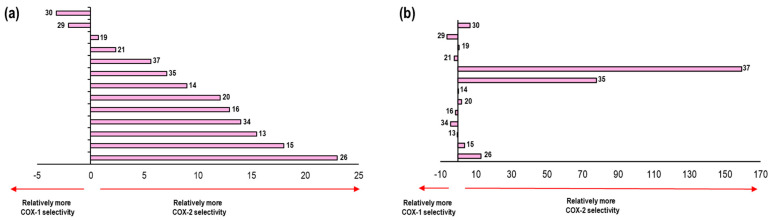
Representation of the selectivity towards one COX isoform. (**a**) Graph representation of selectivity ratio of both isoforms with respect to the calculated values obtained in 1EQG and 4PH9 crystals. E_COX-1_–E_COX-2_ (Kcal/mol) is represented in the X axes (**b**) Graph representation of selectivity ratio of both isoforms with respect to the biology activity values (in vitro) in the 13 compounds. IC_50 COX-1_–IC_50 COX-2_ values (µM) are represented in the X axes.

As the last point analyzed in this system, we carried out a comparison of the energetic values obtained in the binding modes previously described with respect to the experimental IC_50_ values and for the thirteen compounds used in the second validation of our model (Figure 15).

Comparing the selectivity aspect predicted by our model with the already known experimental values of the thirteen compounds, we found a qualitative correlation in the following eight compounds: **29**, **19**, **37**, **35**, **14**, **20**, **15**, and **26**. Seven of these showed selectivity towards the COX-2 isoform, while only compound **29** maintained selectivity towards COX-1.

- The Indomethacin (2OYU and 4COX)

**Figure 16:**
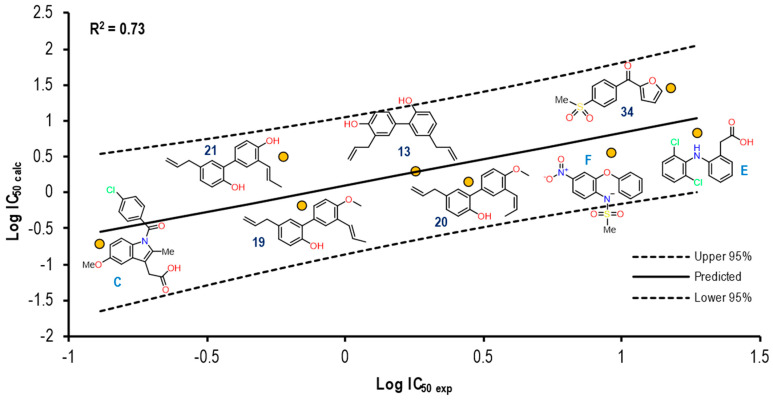
Graphical representation of Log IC_50 calc_ vs Log IC_50 exp_. The 2D structures of molecules that fit in the COX-1 crystal (2OYU) model. Dotted lines show the confidence interval based on the standard deviation.

Continuing with the second pair of crystals, Figure 16 shows the graph with the values of Log IC_50 exp_ (X-axis) and Log IC_50 calc_ (Y-axis), as well as the R^2^ = 0.73. The molecules shown in the graph are those in which it was possible to correlate the $E_{int}$ with their experimental IC_50_ by means of the IC_50_ obtained from the mathematical model that correlates $E_{int}$ obtained from the docking in the 2OYU crystal (see Figure S14).

For the 2OYU crystal, a reproducibility of eight molecules out of **21** was achieved, observing that all structures present two aromatic rings, HBD and HBA. The Indomethacin and **13** showed a position close to the prediction line in comparison with **21** and **34**. Although the co-crystallized Indomethacin alpha is a large structure, the binding mode was not favored for molecules of a similar size, such as the coxibs.

The $E_{int}$*,* experimental and calculated Log IC_50_ and Δerror (Log IC_50 calc_–Log IC_50 exp_) values are shown in Table S4.

**Figure 17:**
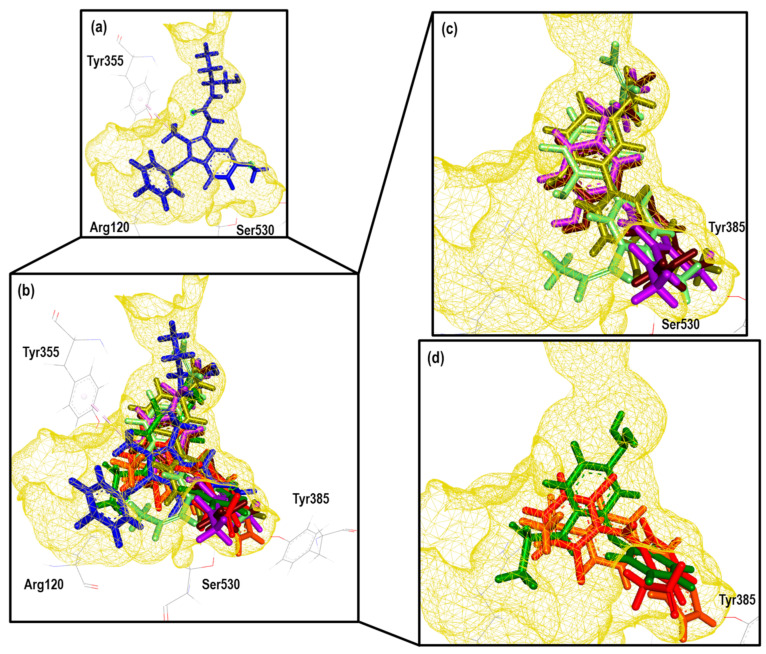
Poses obtained from the docking calculation of the 2OYU crystal. (**a**) Indomethacin is the color blue. (**b**) Posee of all the ligands that fit the 2OYU model; ligands are colored in the following form: 21 (olive green), 19 (purple), 13 (lilac stick), 20 (aquamarine), 34 (orange), Diclofenac (red), Nimesulide (green). (**c**) Ligands 13, 19, 20 and 21 binding modes (**d**) Ligands 34, Diclofenac and Nimesulide binding modes.

To further analyze the relationship between the values obtained from Figure 13 and Table S4, we examined the binding of the eight ligands in the cavity of the 2OYU crystal for COX-1. In Figure 17, all the ligands shown in Figure 16 are displayed in the cavity of COX-1.

We did not detect a close relationship between the size and shape of the cavity with the ligands that were docked into it. Reflecting this, the size of the cavity was clearly affected by the size of the ligand. COX-1 of the 2OYU crystal has a cavity volume of 288.76 Å^3^. Most of the molecules are smaller than Indomethacin alfa. However, the size and shape of the binding site were not limited with respect to the type of molecule being docked. We did not find molecules belonging to the coxibs family or their derivatives.

**Figure 18:**
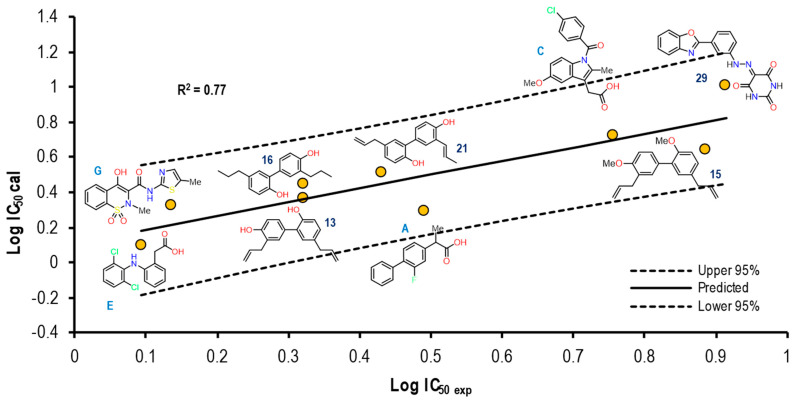
Graphical representation of Log IC_50 calc_ vs Log IC_50 exp_. The 2D structures of molecules that fit in the COX-2 crystal (4COX) model. Dotted lines show the confidence interval based on the standard deviation.

Figure 18 shows the graph with the values of Log IC_50 exp_ (X axis) and Log IC_50 calc_ (Y axis), as well as R^2^ = 0.77. The molecules shown in the graph are those in which it was possible to correlate the $E_{int}$ with their experimental IC_50_ by means of the IC_50_ obtained from the mathematical model that correlates $E_{int}$ obtained from the docking in the 4COX crystal (see Figure S15).

In the case of the 4COX crystal, a reproducibility of nine molecules out of 21 was achieved, observing greater structural diversity in this crystal. The structures present two and three aromatic rings, HBD and HBA. The Indomethacin and **13** showed a position close to the prediction line in comparison with Flurbiprofen and **29**. Finally, structures with smaller sizes showed a higher IC_50 calc_ accuracy with respect to the IC_50 exp_.

The $E_{int}$*,* experimental and calculated Log IC_50_ and Δerror (Log IC_50 calc_–Log IC_50 exp_) values are shown in Table S5.

**Figure 19:**
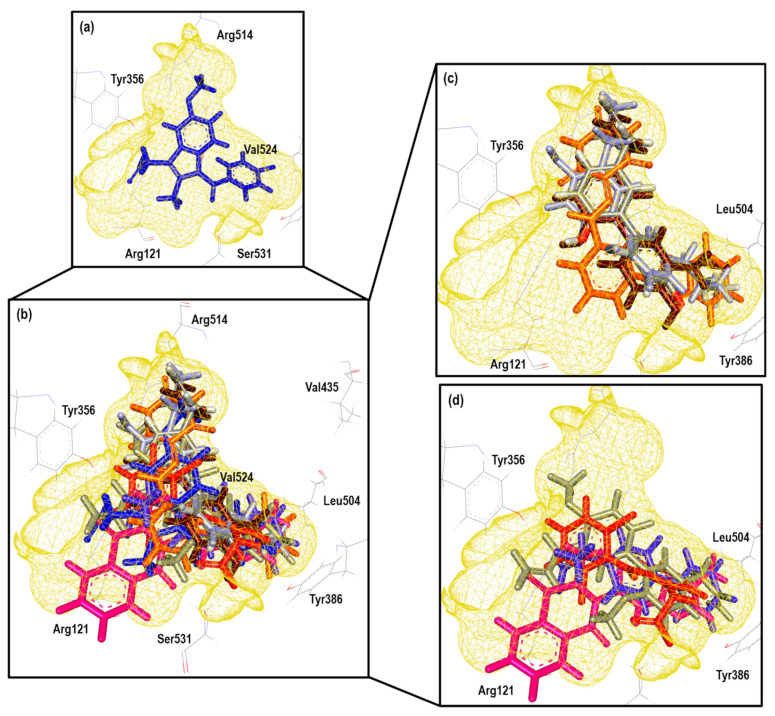
Poses obtained from the docking calculation over 4COX crystal. (**a**) Indomethacin is the color blue. (**b**) Poses of all the ligands that fit the 4COX model; ligands are colored in the following form: 16 (blue gray), 21 (olive green), 15 (stick), 13 (lilac), Flurbiprofen (blue dark), Diclofenac (red), Meloxicam (Magenta) and 29 (yellow). (**c**) Ligands 13, 16, 21, and 29 binding modes. (**d**) Ligands 15, Diclofenac, Flurbiprofen and Meloxicam binding modes.

To further analyze the relationship between the values obtained from Figure 18 and Table S5, we examined the binding of the eight ligands in the cavity of the 4COX crystal for COX-2. In Figure 19, all the ligands shown in Figure 18 are displayed in the cavity of COX-2.

Figure 19 shows the binding mode of the nine ligands in the system of the Indomethacin crystal (4COX) of COX-2 (cavity), as well as the characteristic residues for COX-2.

We detected a close relationship between the size and shape of the cavity with the ligands that were docked into it. COX-2 of the 4COX crystal has a cavity volume of 260.09 Å^3^. Observing the binding modes of **13**, **16**, **21**, and **29**, we noted an accommodation between the hydrophilic pockets BII and BIII. In the case of **15**, Diclofenac, Flurbiprofen, and Meloxicam maintained a horizontal position between pockets BI and BIII. In this crystal, we found that the size and shape of the binding site were not limited with respect to the type of molecule being docked. We find molecules belonging to the coxibs family and their derivatives, as well as other NSAIDs.

**Figure 20:**
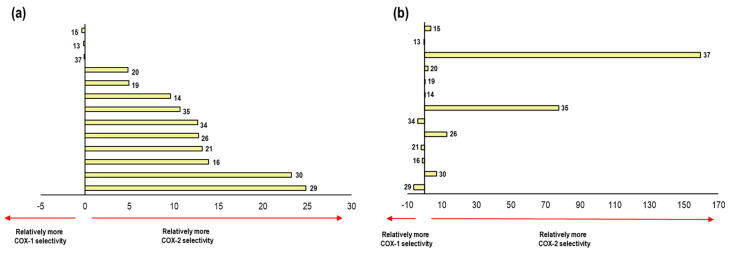
Representation of the selectivity towards one COX isoform. (**a**) Graph representation of selectivity ratio on both isoforms with respect to the calculated values obtained in 2OYU and 4COX crystals. E_COX-1_–E_COX-2_ (Kcal/mol) is represented by the X axes (**b**) Graph representation of selectivity ratio on both isoforms with respect to the biology activity values (in vitro) for the 13 compounds. IC_50 COX-1_–IC_50 COX-2_ values (µM) is represented in the X axes.

Figure 20 shows the comparison of the selectivity obtained by our model in 2OYU and 4COX crystals with the experimental data for the 21 compounds.

We compared the selectivity obtained by our model with experimental data for this system. For the ligands, **15**, **13**, **20**, **19**, **14**, **35**, **26**, and **30** showed a positive correlation in selectivity with a qualitative prediction of 8 molecules out of 13.

- The Celecoxib case (3KK6 and 3LN1)

**Figure 21:**
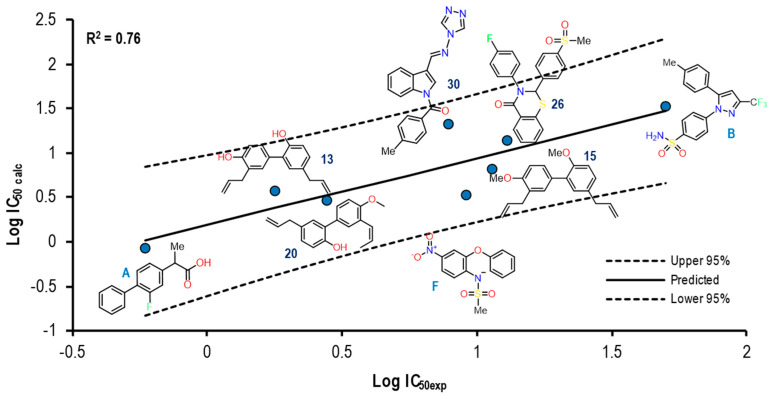
Graphical representation of Log IC_50 calc_ vs Log IC_50 exp_. The 2D structures of molecules that fit in the COX-1 crystal (3KK6) model. Dotted lines show the confidence interval based on the standard deviation.

Figure 21 shows the graph with the values of Log IC_50 exp_ (X axis) and Log IC_50 calc_ (Y axis) as well as R^2^ = 0.76. The molecules shown in the graph are those in which it was possible to correlate $E_{\mathrm{int}}$ with their experimental IC_50_ by means of the IC_50_ obtained from the mathematical model that correlates $E_{int}$ obtained from the docking in the 3KK6 crystal (see Figure S16).

With a total of eight molecules within the experiment, where the compounds Flurbiprofen, **20**, **26** and the ligand itself corresponding to this crystal (Celecoxib) present a closeness to the line (mean), indicating a good correlation between these two values, the opposite case was observed in Nimesulide and compound **30**, and the rest of the molecules of this experiment showed a similar behavior. Finally, when comparing the structures, we found that at least 50% presented a similar size to Celecoxib, which was an important factor in delimiting the size and shape of the cavity present in this crystal, allowing larger molecules to carry out the interaction with the binding site. In addition, this group of structures presents a lower IC_50_ value towards the COX-2 isoform, indicating a higher selectivity to it.

The $E_{int}$, experimental and calculated Log IC_50_ and Δerror (Log IC_50 calc_–Log IC_50 exp_) values are shown in Table S6.

To further analyze the relationship between the values obtained from Figure 21 and Table S6, we examined the binding of the nine ligands in the cavity of the 3KK6 crystal for COX-1.

**Figure 22:**
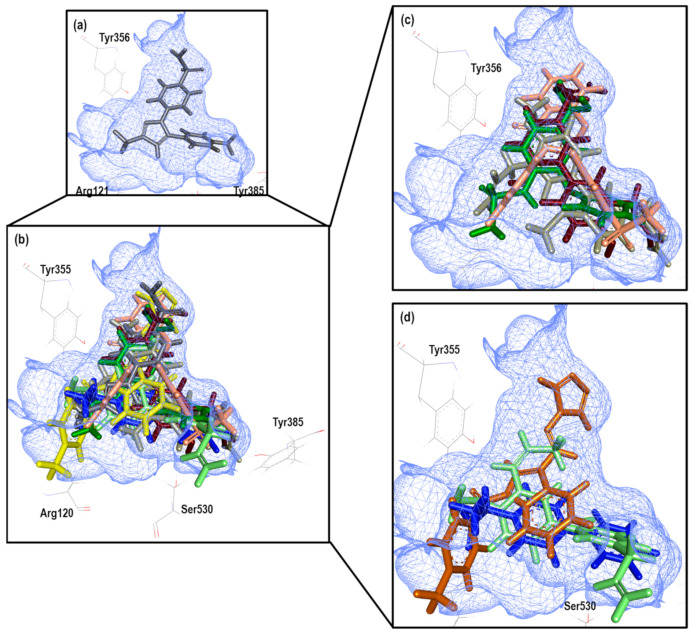
Poses obtained from the docking calculation over 3KK6 crystal. (**a**) Celecoxib is the color gray. (**b**) Poses of all the ligands that fit the 3KK6 model; ligands are colored in the following form: 13 (lilac), 20 (aquamarine), 15 (wine), Nimesulide (green), 26 (salmon pink), 30 (brown light), Flurbiprofen (blue dark). (**c**) Ligands 13, 15, 26 and Nimesulide binding modes. (**d**) Ligands 20, 30 and Flurbiprofen binding modes.

Figure 22 shows the binding mode of the eight ligands in the system of Celecoxib (3KK6) of COX-1 (cavity), as well as the characteristic residues for COX-1.

At least 6 of the 8 molecules, with the obviating structure of Celecoxib as our reference ligand, presented a binding mode that was quite similar with respect to the spatial arrangement; the arrangement had a tendency towards the upper cavity of the binding site, where the sulfonamide group of the NSAIDs of the COXIBs family was hosted. The exception was Flurbiprofen, which maintained its binding mode by orienting with the carboxylic acid group in the direction of Arg120 and the benzene ring towards Tyr385.

**Figure 23:**
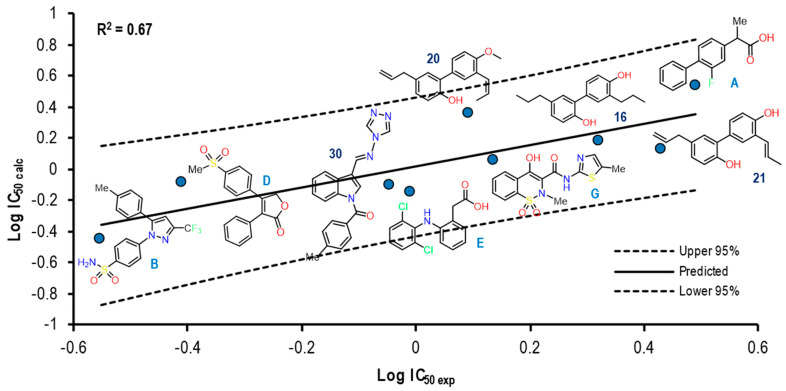
Graphical representation of Log IC_50 calc_ vs Log IC_50 exp_. The 2D structures of molecules that fit in the COX-2 crystal (3LN1) model. Dotted lines show the confidence interval based on the standard deviation.

Figure 23 shows the graph with the values of Log IC_50 exp_ (X-axis) and Log IC_50 calc_ (Y-axis), as well as R^2^ = 0.67. The molecules shown in the graph are those in which it was possible to correlate the $E_{int}$ with their experimental IC_50_ by means of the IC_50_ obtained from the mathematical model that correlates $E_{int}$ obtained from the docking in the 3LN1 crystal (see Figure S17).

This graph shows how five of the nine total molecules in the experiment present a proximity to the line (mean), indicating a good correlation between these two values and how the rest of the molecules (4) denote a greater distance; however, the nine molecules are within the statistical limits without presenting any outlier. In this group of molecules, we found NSAIDs with greater selectivity towards the COX-2 isoform, such as Rofecoxib (coxibs), Meloxicam (oxicams), and Diclofenac, as well as structures **30** and **20** with lower IC_50_ values for COX-2.

The $E_{int}$, experimental and calculated Log IC_50_ and Δerror (Log IC_50 calc_–Log IC_50 exp_) values are shown in Table S7.

To further analyze the relationship between the values obtained from Figure 23 and Table S7, we examined the binding of the nine ligands in the cavity of the 3LN1 crystal for COX-2.

**Figure 24:**
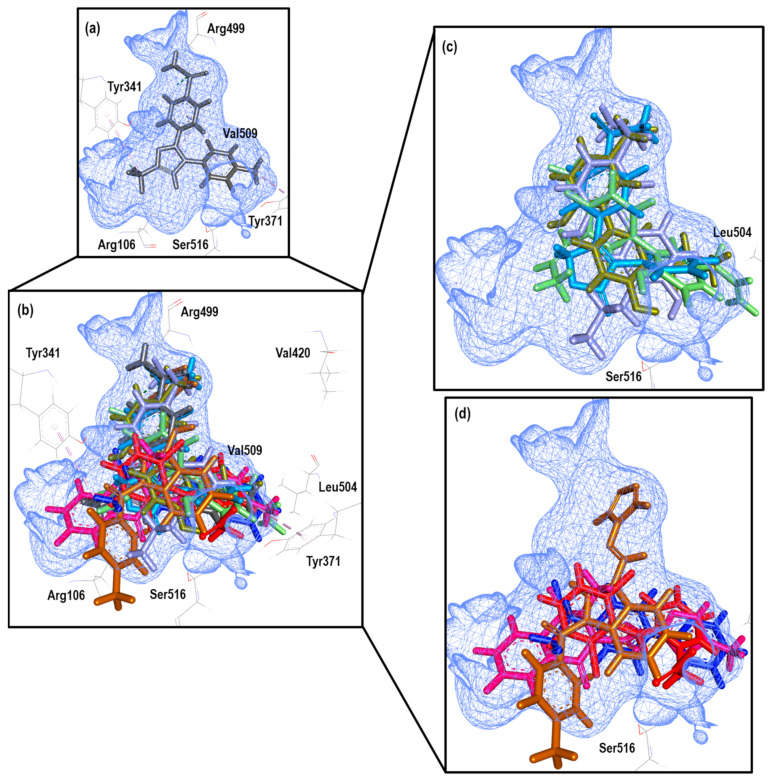
Poses obtained from the docking calculation over 3LN1 crystal. (**a**) Celecoxib is the color gray. (**b**) Poses of all the ligands that fit the 3LN1 model; ligands are colored in the following form: 21 (olive green), 16 (blue gray), 20 (aquamarine stick), Diclofenac (red), Rofecoxib (blue light), Flurbiprofen (blue dark), 30 (brown) and Meloxicam (Magenta). (**c**) Ligands 16, 20, 21 and Rofecoxib binding modes. (**d**) Ligands 30, Diclofenac, Flurbiprofen and Meloxicam binding modes.

Figure 24 shows the binding mode of the eight ligands in the system of Celecoxib (3LN1) of COX-2 (cavity), as well as the characteristic residues for COX-2.

In this experiment, we observed how structure **30** presents a mode like that observed in the COX-1 crystal, but which is different in the 4PH9 crystal despite belonging to the COX-2 isoform; however, the value of the Δ error is lower in this system. On the other hand, known NSAID structures present the binding mode according to the already known one. Finally, within the structures for which a binding mode has not been established, a tendency for accommodation in the direction of Arg499 was observed.

**Figure 25:**
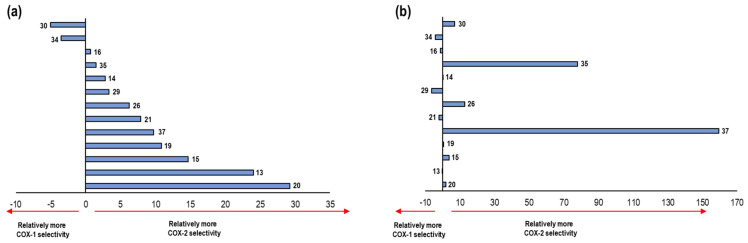
Representation of the selectivity towards one COX isoform. (**a**) Graph representation of the selectivity ratio on both isoforms with respect to the calculated values obtained in 3KK6 and 3LN1 crystals. E_COX-1_−E_COX-2_ (Kcal/mol) is represented by the X axes (**b**) Graph representation of the selectivity ratio on both isoforms with respect to the biology activity values (in vitro) in the 13 compounds. IC_50 COX-1_−IC_50 COX-2_ values (µM) are represented in the X axes.

As a final part of the analysis in the Celecoxib system, we compared the selectivity-energy values obtained for each of the ligands in both crystals (3KK6 and 3NL1) according to our model. These data were compared with the experimental selectivity values, as shown in the following figure (Figure 25).

In relation to the values obtained in our model. there was a tendency towards selectivity for COX-2 for most of the compounds; however, of the 13 compounds, 8 were able to reproduce the selectivity aspect of the Celecoxib system (3KK6 and 3LN1) in comparison with the experimental data. The compounds that obtained qualitative selectivity values were **34**, **35**, **14**, **26**, **37**, **19**, **15** and **20**.

- The Meloxicam case (4O1Z and 4M11)

**Figure 26:**
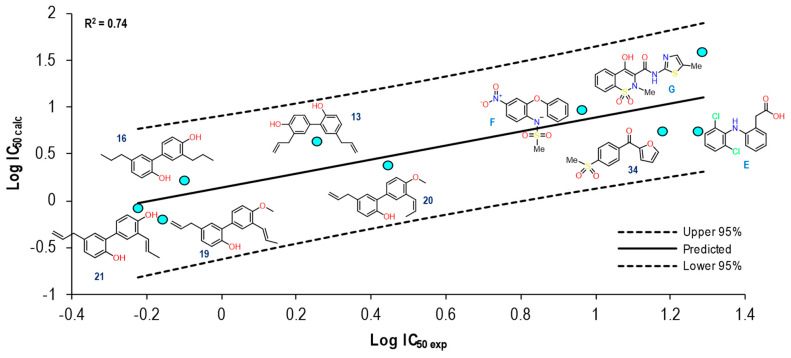
Graphical representation of Log IC_50 calc_ vs Log IC_50 exp_. The 2D structures of molecules that fit in the COX-1 crystal (4O1Z) model. Dotted lines show the confidence interval based on the standard deviation.

Figure 26 shows the graph with the values of Log IC_50 exp_ (X axis) and Log IC_50 calc_ (Y axis), as well as R^2^ = 0.74. The molecules shown in the graph are those in which it was possible to correlate the $E_{int}$ with their experimental IC_50_ by means of the IC_50_ obtained from the mathematical model that correlates $E_{int}$ obtained from the docking in the 4O1Z crystal (see Figure S18).

**Figure 27:**
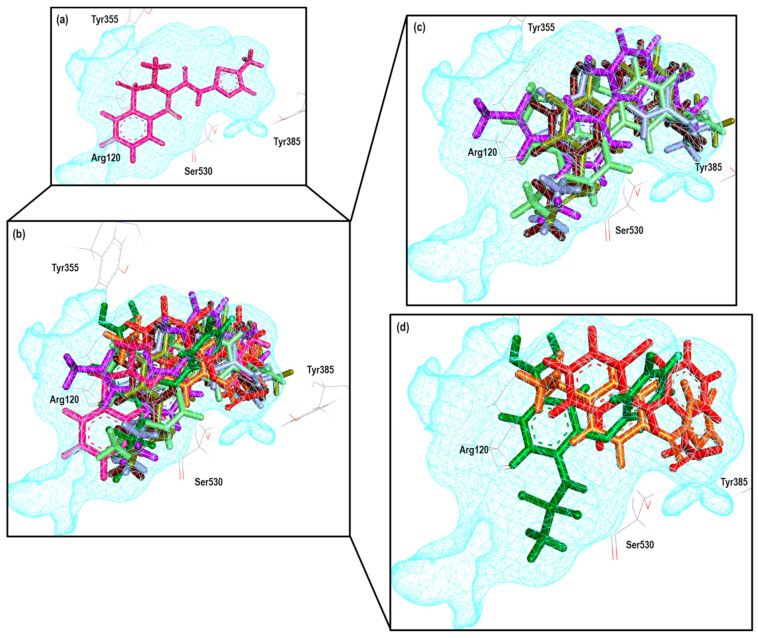
Poses obtained from the docking calculation over 4O1Z crystal. (**a**) Meloxicam is the color magenta. (**b**) Poses of all the ligands that fit the 4O1Z model; ligands are colored in the following form: 13 (lilac), 21 (olive green), 16 (blue gray), 19 (purple), 20 (aquamarine), Diclofenac (red), Nimesulide (green) and 34 (orange). (**c**) Ligands 13, 16, 19, 20 and 21 binding modes. (**d**) Ligands 34, Diclofenac and Nimesulide binding modes.

Reproducibility was achieved for nine total molecules, with compound **13** and Meloxicam being the farthest from the line (mean) within the graph; the rest of the molecules exhibited similar behavior except for compound 21, which showed the closest proximity to the line (mean). The $E_{int}$, experimental and calculated Log IC_50_ and Δerror (Log IC_50 calc_−Log IC_50 exp_) values are shown in Table S8. To further analyze the relationship between the values obtained from Figure 26 and Table S8, we examined the binding of the nine ligands in the cavity of the 4O1Z crystal for COX-1. Figure 27 shows the binding mode of the eight ligands in the system of Meloxicam (4O1Z) of COX-1 (cavity), as well as the characteristic residues for COX-1.

We can observe that compounds like 13 can change the binding mode depending on the crystal in which the experiment is carried out, incorporating the molecules in the molecules in the specific cavity of the NSAIDs of the oxicams type. Although the value of the Δ error calculated for Meloxicam is presented as one of the highest values in this experiment, we were able to demonstrate that the binding mode of this structure is being carried out properly.

**Figure 28:**
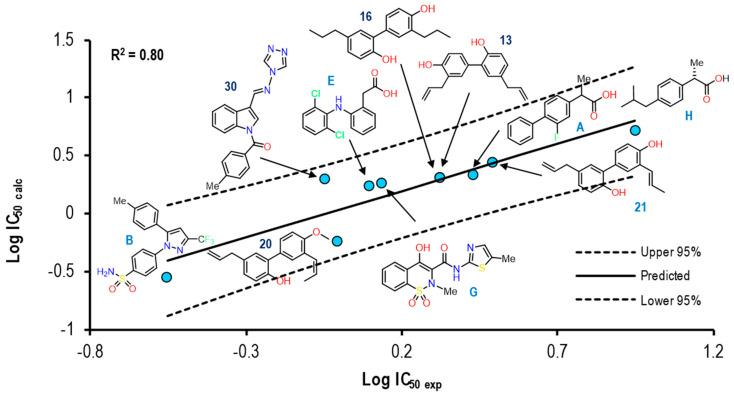
Graphical representation of Log IC_50 calc_ vs Log IC_50 exp_. The 2D structures of molecules that fit in the COX-2 crystal (4M11) model. Dotted lines show the confidence interval based on the standard deviation.

Figure 28 shows the graph with the values of Log IC_50 exp_ (X axis) and Log IC_50 calc_ (Y axis), as well as R^2^ = 0.80. The molecules shown in the graph are those in which it was possible to correlate the $E_{int}$ with their experimental IC_50_ by means of the IC_50_ obtained from the mathematical model that correlates with $E_{int}$ obtained from the docking in the 4M11 crystal (see Figure S19).

**Figure 29:**
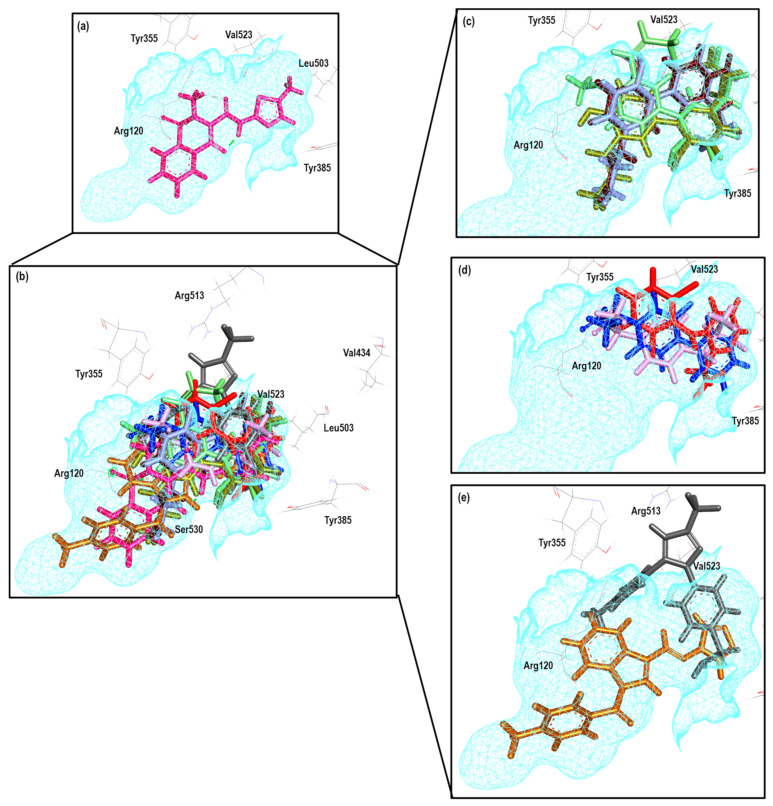
Poses obtained from the docking calculation over 4O1Z crystal. (**a**) Meloxicam is the color magenta. (**b**) Poses of all the ligands that fit the 4O1Z model; ligands are colored in the following form: 13 (lilac), 16 (blue gray), 21 (olive green), 20 (aquamarine), 30 (brown), Celecoxib (grey), Diclofenac (red), Flurbiprofen (blue dark), Ibuprofen (pink). (**c**) Ligands 13, 20, 21, 16 and binding modes. (**d**) Ligands Ibuprofen, Flurbiprofen and Diclofenac binding modes. (**e**) Ligands Celecoxib and 30 binding modes.

In comparison with all the crystals, this was one that managed to reproduce more molecules, finding structures of the non-selective type to be selective. Only compounds **30** and **20** showed a greater distance from the line (mean), while compounds **16**, **13**, and **21** obtained better IC_50_ correlation values. None of the ten structures were outside the statistical confidence intervals. The $E_{int}$, experimental and calculated Log IC_50_ and Δerror (Log IC_50 calc_ − Log IC_50 exp_) values are shown in Table S9. To further analyze the relationship between the values obtained from Figure 28 and Table S9, we examined the binding of the nine ligands in the cavity of the 4O1Z crystal for COX-1. Figure 29 shows the binding mode of the eight ligands in the system of Meloxicam (4M11) of COX-2 (cavity), as well as the characteristic residues for COX-2.

It should be noted that the binding mode presented by Celecoxib in this crystal is not the correct one; despite the accurate prediction of the Log IC_50 calc_ value, this does not agree with the binding mode. However, in the case of Ibuprofen and Flurbiprofen, they did present the proper binding mode where both directed the carboxylic acid group to the Arg120 residue and the hydrophobic section with an orientation towards Leu503 in a horizontal position. In the case of structure **30**, the ρ-MeBn ring was incorporated into the specific cavity of the oxicams.

**Figure 30:**
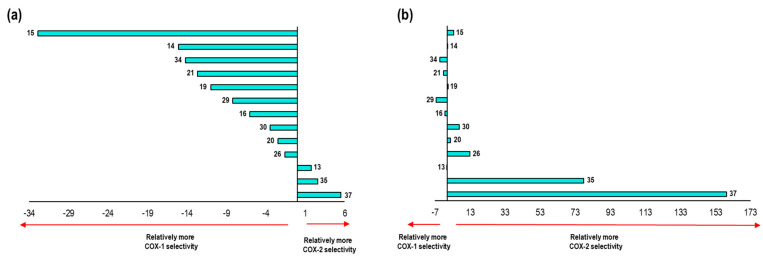
Representation of the selectivity towards one COX isoform. (**a**) Graph representation of the selectivity ratio of both isoforms with respect to the calculated values obtained in 4O1Z and 4M11 crystals. E_COX-1_–E_COX-2_ (Kcal/mol) is represented in the X axes (**b**) Graph representation of the selectivity ratio of both isoforms with respect to the biology activity values (in vitro) in the 13 compounds. IC_50 COX-1_–IC_50 COX-2_ values (µM) is represented in the X axes.

At this point, we already obtained the predicted IC_50_ values as well as the binding modes for the compounds in the Meloxicam system (4O1Z and 4M11). We only needed to compare the selectivity for these compounds observed in this system with respect to the known experimental values (Figure 30).

A tendency towards selectivity for COX-1 was observed for most of the compounds; however, of the three compounds, four were able to reproduce the selectivity aspect for the Meloxicam system (4O1Z and 4M11) in comparison with the experimental data. The compounds that obtained qualitative selectivity values were as follows: **34**, **21**, **29**, and **16**.

### 2.6. Structural Analysis of Inhibitors from COX-1 and COX-2

**Figure 31:**
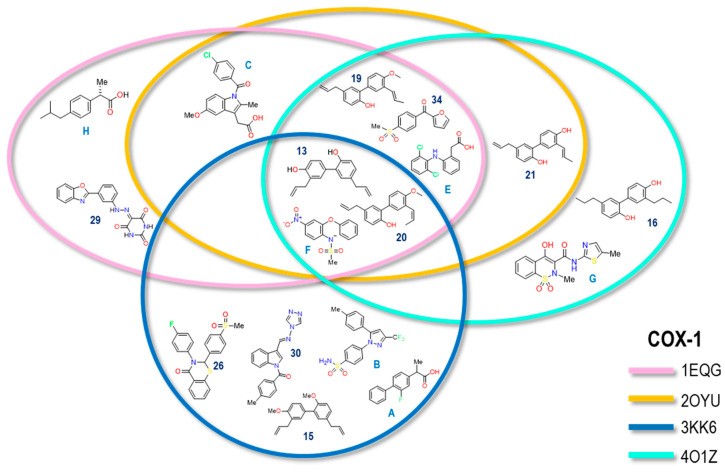
The Venn diagram illustrates the correlation between experimental and calculated IC_50_ values of molecules associated with the COX-1 crystal. The crystals included are as follows: 1EQG (Ibuprofen, pink), 2OYU (Indomethacin, yellow), 3KK6 (Celecoxib, blue), and 4O1Z (Meloxicam, cyan).

The reproducibility of Log IC_50 exp_ compared to Log IC_50 calc_ for the molecules in each of the COX crystals was assessed. Figure 31 shows a Venn diagram with each ring representing one of the crystals used in the COX-1 analysis. The location of the structures corresponds to the crystal or crystals where the reproducibility of the calculated vs experimental IC_50_ values was carried out.

The data extracted from the Venn diagram reveal that three molecules (13, 20, and Nimesulide) exhibited reproducibility in terms of energy compared to the experimental IC_50_ values across all four crystals. These molecules shared structural features, such as two aromatic rings, and possessed hydrogen bond acceptor (HBA) and hydrogen bond donor (HBD) properties. Similarly, three molecules (19, 34, and Diclofenac) displayed reproducibility in the 1EQG, 2OYU, and 4O1Z crystals. Like the previous group, these molecules also featured two aromatic rings, including HBA and HBD properties. Notably, Diclofenac and **19** showed a slight preference for isoform 2 in terms of selectivity. However, they did not demonstrate reproducibility in the Celecoxib crystal. For the case of Indomethacin, its reproducibility was only achieved on 1EQG and 2OYU crystals, the latter being the structure with which the ligand was co-crystallized; this crystal has a bigger cavity caused by the structural increment in the Indomethacin alpha.

On the contrary, compound **21** shared reproducibility in the 2OYU and 4O1Z crystals. Despite its structural similarity to other compounds, we did not observe its correlation with other crystals. In the case of Meloxicam’s structure, reproducibility occurred in the same crystal as in the case of Ibuprofen and Celecoxib. An unexpected outcome was the reproducibility of structure **29** on the ibuprofen crystal since this is a molecule with a larger size and functional groups different from those of ibuprofen. Finally, among the five molecules that only reproduced in the 3KK6 crystal, three shared a similar size with Celecoxib. This suggests that, in crystals where the cavity size is predetermined by the co-crystallized ligand’s size, the likelihood of reproducing molecules of the same size or larger is higher.

**Figure 32:**
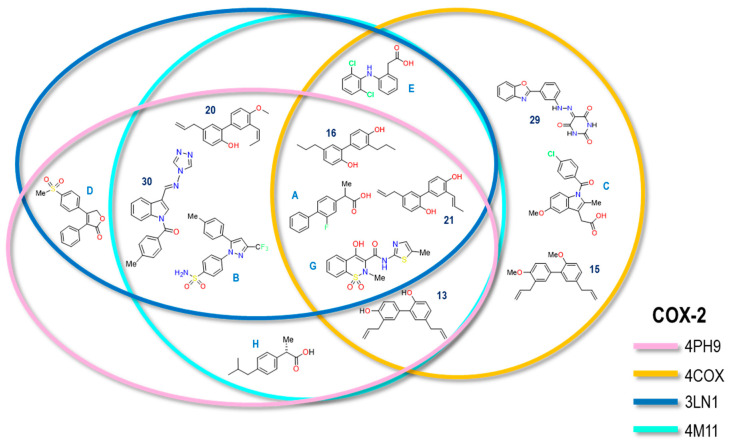
The Venn diagram illustrates the correlation between experimental and calculated IC_50_ values of molecules associated with the COX-2 crystal. The crystals included are as follows: 4PH9 (Ibuprofen, pink), 4COX (Indomethacin, yellow), 3NL1 (Celecoxib, blue), and 4M11 (Meloxicam, cyan).

The same analysis was carried out for the COX-2 crystals. Figure 32 shows the Venn diagram where each of the rings represents one of the crystals used in the COX-2 analysis. The structures are located based on the crystal or crystals where we compared the reproducibility of the calculated and experimental values.

In the case of COX-2 crystals, the diagram shows four molecules (Meloxicam, Flurbiprofen, 16 and 21) that were reproducible in terms of energy vs. experimental IC_50_ in the four crystals; likewise, in the case of diclofenac, it achieved reproducibility in three of the four crystals (4COX, 3LN1, and 4M11). In the case of Celecoxib, reproducibility was observed in crystals 4PH9, 3LN1, and 4M11, as well as for compounds **30** and **20**; it should be noted that molecule **30** is even larger than Celecoxib itself. On the other hand, Rofecoxib and Ibuprofen were only found in two of the four crystals, including 4PH9 and 3LN1 in the first case and 4PH9 and 4M11 in the second. Finally, Indomethacin can be observed in the diagram as it is only found within the same crystal with compounds **29** and **15**, which were only found in one (4COX), limiting the reproducibility to a single crystal. As in COX-1, compounds of the COXIBs type were only able to experience reproducibility in their own crystal, as in 4M11 and 4PH9.

## 3. Materials and Methods

### 3.1. Search and Selection of PDBs

COX crystals were obtained from the Protein Data Bank [21] and were selected based on optimal resolution levels. The chosen PDB entries include 1EQG (2.6 Å), 2OYU (2.7 Å), 3KK6 (2.75 Å), and 4O1Z (2.4 Å) for COX-1 from the Ovis aries organism, and 4PH9 (1.81 Å), 4COX (2.9 Å), 3NL1 (2.15 Å), and 4M11 (2.45 Å) for COX-2 from the Mus Musculus organism, employing the X-ray diffraction crystallization technique.

Additionally, we assessed structural quality elucidation (EDIA) by calculating the weighted sum over all relevant grid points in the sphere of interest surrounding the atom. The sphere’s radius was twice the resolution-dependent electron density sphere radius [22]. This evaluation tool is accessible on the Protein Pluss server [23].

Sequence alignment was performed with the Clustal Omega server [24]. The sequences were obtained from the Fasta file of the corresponding PDBs, for the case of the Ovis aries sequence for COX-2 and Mus musculus for COX-1 [25].

To initiate the construction of the prediction model, the first step involved searching for crystals of COX 1 and 2 enzymes available in the Protein Data Bank database. A total of 49 crystals from the organisms Ovis aries (COX-1) and Mus Musculus (COX-2), each with distinct co-crystallized ligands, were obtained (Figure S20).

After locating these crystals, we analyzed their quality using X-ray crystallization techniques. The evaluation considered the reported resolution value for each crystal, excluding those with values greater than 3 Å. Subsequently, crystals meeting this criterion were categorized based on the characteristics of the co-crystallized ligand, including structure, size, binding mode, and selectivity (Figure S21).

Based on the cocrystallized ligand family, four distinct groups were derived as follows: endogenous inhibitors, propionic acid derivatives, heterocyclic biaryl NSAIDs (COXIBs), and enolic acid derivatives (oxicams); nevertheless, the number of crystals remained significantly high.

Therefore, we conducted a thorough assessment of the crystallographic technique’s quality. The evaluation involved elucidating the crystals, and their resolution was analyzed using the EDIA tool accessible on the Protein Pluss server. This analysis enabled a comparison of the resolution quality in both crystals concerning the electron density for each atom, depicted by the blue color on the scale. Those atoms with minimal inconsistencies in electron density distribution are highlighted in Figure S22 [26]. Subsequently, having obtained the crystals of the highest quality, we proceeded with the selection based on ligand characteristics, including structure, size, binding mode, and selectivity.

We found that the endogenous ligand presented a similar binding mode to the oxicams. However, this region was different from NSAIDs such as Ibuprofen and Celecoxib. For this reason, we decided to exclude this crystal from our group and incorporate both COX crystals with the Indomethacin ligand into our selection since this NSAID was utilized previously in the in vivo assays of the molecules synthesized within our research group, besides being one of the selective NSAIDs for COX-1, forming part of the controls for our computational model.

As a result, we integrated our model with a total of eight final crystals. For each one of the COXs, four crystals were selected with the following ligands: Ibuprofen, Indomethacin, Celecoxib, and Meloxicam, as shown in Figure S23.

### 3.2. Search for In Vitro Biological Activity Values on Previously Reported NSAIDs and New Compounds (IC_50_)

The search for biological in vitro activity on reported NSAIDs and new compounds was realized in the database ChEMBL, which is a manually curated database of bioactive molecules with drug-like properties. It brings together chemical, bioactivity, and genomic data to aid the translation of genomic information into effective new drugs [27].

The values of IC_50_ employed in this model were considered with the following criteria in the database: IC_50_ values with undefined concentration ranges were not taken into account for the model; the range of IC_50_ values had to be between 0.1 µM and 159.72 µM from COX-1 and 0.05 µM to 19.2 µM from COX-2 in vitro models, and the tests were performed on kits of the inhibition of ovine COX-1 and 2 via enzyme immunoassay [17,28,29,30,31,32].

### 3.3. Construction and Structural Analysis of Ligands

Therefore, we carried out the full geometric optimization without symmetric restriction and vibrational frequency calculations on the NSAIDs and anti-inflammatory compounds (neutral or anions) at a Semi-Empirical level using the Parametric Method 6 (PM6) approximation. From these calculations, the Mulliken partial charge were obtained; this type of partial charges was chosen based on previous works [33,34]. All the calculations were made in SPARTAN 20 [35].

### 3.4. Molecular Docking Calculations Docking Methodology

Molecular docking calculations were carried out in Molegro Virtual Docker 6.0 [36], employing the crystals retrieved from the Protein Data Bank (vide supra). For COX-1 (PDB: 1EQG) [37], [2OYU] [38], (3KK6) [39], (4O1Z) [40] and for COX-2 (PDB: 4PH9) [41], (4COX) [42], (3NL1) [43], and (4M11) were used [40]. Each crystal was complexed with an NSAID: Ibuprofen (1EQG and 4PH9), Indomethacin (2OYU and 4COX), Celecoxib (3KK6 and 3LN1) and Meloxicam (4O1Z and 4M11). The potential binding sites (defined as cavities) of both COX-1 and COX-2 were detected using the expanded Van der Waals spheres method. The cavities found for COX-1 PDB: 1EQG (23.04 Å^3^), 2OYU (112.64 Å^3^), 3KK6 (91.64 Å^3^), 4O1Z (76.8 Å^3^) and COX-2 PDB: 4PH9 (56.32 Å^3^), 4COX (52.22 Å^3^), 3NL1 (84.48 Å^3^), 4M11 (111.61 Å^3^), where all the binding calculations were performed, corresponded to the binding site of each ligand in both isoforms (henceforth known as the binding site). All water molecules were removed from the crystal.

Rigid and flexible docking was performed. For the flexible approach, the residues within 4 Å from the reference ligands (i.e., Ibuprofen, Indomethacin, Celecoxib, Meloxicam) were set as flexible; the type and number of residues are shown in Table S10 for COX-1 and COX-2. The docking calculation was performed for both types of compound, NSAID and biological compounds, with their corresponding active enantiomer. Partial charges were set according to Molegro Virtual Docker’s internal partial charges scheme and the Mulliken partial charges scheme. All the residues bearing four free-rotating bonds were assigned with no strength factor value limiting their movement. For those residues with three rotational bonds, a value of 0.75 was assigned, and 0.5 for those with two free-rotating bonds. The search function MolDock Optimizer was employed for COX-1 and COX-2, and both functions used the genetic algorithm technique for the search for the best binding mode of the given compound. For calculating the binding energy, the scoring function Moldock Score [GRID] was used. For the scoring function, the search sphere was fixed with a 14 Å radius value, and a GRID partition of 0.2 Å was set. The other parameter was used as 2000 minimization steps for each flexible residue, and the ligand with 2000 steps of global minimization per run. For the energetic analysis of the ligand, the electrostatic internal interactions, the internal H-bond and the sp^2^-sp^2^ torsions were considered. For the MolDock SE function of all the crystal, a total of 10 run with 1500 iterations, and a population of 50 individuals per run was used, with the exception of 4M11, where the MolDock SE algorithm -with a total of 10 runs, 3000 iterations, and a population of 50 individuals per run- was used. For the docking flex of COX-1, the Moldock optimizer algorithm—with a total of 10 runs, 2000 iterations, a population of 50 individuals per run—was used from 1EQG, 2OYU, and 3KK6. With respect to the selection of poses, the automorphism option handled by the program was considered. In the case of 4O1Z, a function total of 15 runs with 2000 iterations and a population of 50 individuals per run was used. All the residue rotating bonds were assigned a 1.0 strength factor for the selection of poses (vide supra).

For the docking flex of COX-2, the Moldock optimizer function with a total of 10 runs 3000 iterations and a population of 50 individuals per run, was considered from 4PH9. From 4COX, a function total of 10 runs with 2000 iterations and a population of 50 individuals per run was used. For 3NL1, a function total of 10 runs with 3000 iterations, and a population of 50 individuals per run was used, and finally, a function total of 15 runs with 2000 iterations and a population of 50 individuals per run was used in the case of 4M11 for the selection of poses (vide supra).

## 4. Conclusions

In this study, we introduced a computational method to improve the precision of predictive values, such as binding energy, the interaction mode, and selectivity, for the interaction of small molecules with both COX isoforms. This achievement resulted from an exhaustive analysis involving various conformations of these enzymes based on their crystallized structures.

The results underscore that the quality of an analysis and, consequently, the accuracy of predictions depend significantly on the selection of crystals because the conformation adopted by the enzyme, and influenced by the co-crystallized ligand, constrains the size and shape of the receptor cavity. For instance, in the case of COXIBs, we noted more challenges in the reproducibility of energy and binding modes when compared to their own crystals.

It suggests that even though some molecules exhibit lower IC_50_ values for COX-2, accurately predicting the binding mode in these crystals is not always feasible.

Regarding Flurbiprofen, a COX-1 selective compound, no energy reproducibility in the Ibuprofen crystal was found, despite both belonging to the same NSAID family. By contrast, in the Meloxicam crystal, we obtained a more consistent correlation in the binding mode, energy values, and selectivity.

Our proposed method implies that, for molecules lacking structural similarity, relying solely on energy values from molecular docking in a single crystal is insufficient. Preferably, an analysis of at least three of these crystals (such as Ibuprofen, Celecoxib, and Meloxicam) with the aim of obtaining energy values that exhibit a similar correlation across all six crystals and a binding mode, as closely resembling as possible, should be conducted. The latter criterion is of paramount importance.

## Figures and Tables

**Table 1:** Evaluation characteristics of the global properties of the crystals used in the computational model and importance of amino acid residues.

PDB	Ligand	Volume (Å^3^)	Surface (Å^2^)	Depth (Å)	Hydrophobicity Ratio	aa in Both Isoforms	aa in Only This Isoform
1EQG (COX-1)	Ibuprofen	204.80	254.18	10.91	0.792	Tyr355, Arg120, Ser530, Tyr385	Ile523, Ile434, Phe503, His513
4PH9 (COX-2)	Ibuprofen	228.86	306.53	11.39	0.760	Tyr356, Arg121, Ser531, Tyr386	Leu504, Val435, Arg514, Val524
2OYU (COX-1)	Indomethacin	288.76	335.33	12.82	0.745	Tyr355, Arg120, Ser530, Tyr385	Ile523, Ile434, Phe503, His513
4COX (COX-2)	Indomethacin	260.09	343.12	12.34	0.780	Tyr355, Arg120, Ser530, Tyr385	Leu503, Val523, Val434, Arg513
3KK6 (COX-1)	Celecoxib	198.14	309.36	10.91	0.763	Tyr341, Arg106, Ser516, Tyr371	Ile523, Ile434, Phe503, His513
3LN1 (COX-2)	Celecoxib	269.31	306.59	12.28	0.760	Tyr355, Arg120, Ser530, Tyr385	Arg513, Leu503, Val523, Val434
4O1Z (COX-1)	Meloxicam	272.89	335.02	12.67	0.815	Tyr355, Arg120, Ser530, Tyr385	Ile523, Ile434, Phe503, His513
4M11 (COX-2)	Meloxicam	292.35	320.25	13.21	0.831	Tyr355, Arg120, Ser530, Tyr385	Leu503, Val384, Val523, Arg513

**Table 2:** Energetic interaction values (Kcal/mol) for NSAIDs in each of the crystals of both isoforms and their delta Energetic (ΔE).

NSAIDs/COX	COX-1				COX-2				ΔE
	1EQG	2OYU	3KK6	4O1Z	4PH9	4COX	3LN1	4M11	
Flurbiprofen	−104.84	−105.12	−93.58	−110.92	−104.45	−123.05	−103.01	−105.91	−5.00
Indomethacin	−155.27	−145.91	−135.67	−152.18	−151.98	−150.60	−146.31	−140.19	−3.29
Ibuprofen	−96.57	−82.19	−79.92	−87.85	−95.66	−109.53	−83.01	−91.59	−0.91
Nimesulide	−122.59	−122.99	−119.00	−104.48	−114.84	−125.41	−117.58	−109.72	2.42
Celecoxib	−168.08	−169.36	−162.36	−128.73	−161.57	−165.60	−169.55	−157.82	7.15
Diclofenac	−108.23	−118.20	−106.68	−108.64	−103.98	−110.47	−114.72	−116.44	7.79
Meloxicam	−109.08	−155.79	−118.00	−92.91	−122.27	−125.28	−135.43	−115.40	17.55
Rofecoxib	−139.99	−135.96	−123.10	−105.56	−140.86	−140.42	−144.84	−111.51	21.74

## Data Availability

Data is contained within the article and Supplementary Materials.

## Supplementary Materials

The following supporting information can be downloaded at: https://www.mdpi.com/article/10.3390/ph16121688/s1.

## References

[1] Clark M.A., Finkel R., Rey J.A., Whalen K. (2012). Antiinflamatorios. Farmacología.

[2] Serhan C.N., Petasis N.A. (2011). Resolvins and Protectins in Inflammation Resolution. Chem. Rev..

[3] Kumar V., Abbas A.K., Aster J.C., Klatt E.C., Kummar R., Mitchell R.N. (2013). Inflamación y reparación. Patología Humana.

[4] Stevens A., Lowe J., Scott I. (2011). Lesión celular y Muerte. Respuestas tisulares al daño. Patología Clínica.

[5] Buch M.H., Eyre S., McGonagle D. (2021). Persistent inflammatory and non-inflammatory mechanisms in refractory rheumatoid arthritis. Nat. Rev. Rheumatol..

[6] Moriya J. (2019). Critical roles of inflammation in atherosclerosis. J. Cardiol..

[7] Ferrer M.D., Busquets-Cortés C., Capó X., Tejada S., Tur J.A., Pons A., Sureda A. (2019). Cyclooxygenase-2 inhibitors as a therapeutic target in inflammatory diseases. Curr. Med. Chem..

[8] Pęczek P., Gajda M., Rutkowski K., Fudalej M., Deptała A., Badowska-Kozakiewicz A.M. (2023). Cancer-associated inflammation: Pathophysiology and clinical significance. J. Cancer Res. Clin. Oncol..

[9] Barreiro E.J., Kümmerle A.E., Fraga C.A.M. (2011). The methylation Effect in Medical Chemistry. Chem. Rev..

[10] Leung C.S., Leung S.S.F., Tirados-Rives J., Jorgensen W.L. (2012). Methylation Effects on Protein-Ligand Binding. J. Med. Chem..

[11] Romero-Estudillo I., Viveros-Ceballos J.L., Cazares-Carreño O., González-Morales A., de Jésus B.F., López-Castillo M., Razo-Hernández R.S., Castañeda-Corral G., Ordóñez M. (2019). 000Synthesis of new α-aminophosphonates: Evaluation as anti-inflammatory agents and QSAR studies. Bioorg. Med. Chem..

[12] Wang B., Wu L., Chen J., Dong L., Chen C., Wen Z., Hu J., Fleming I., Wang D.W. (2021). Metabolism pathways of arachidonic acids: Mechanisms and potential therapeutic targets. Signal Transduct. Target Ther..

[13] Ahmadi M., Bekeschus S., Weltmann K.D., von Woedtke T., Wende K. (2022). Non-steroidal anti-inflammatory drugs: Recent advances in the use of synthetic COX-2 inhibitors. RSC Med. Chem..

[14] Blobaum A.L., Marnett L.J. (2007). Structural and functional basis of cyclooxygenase inhibition. J. Med. Chem..

[15] Volkamer A., Griewel A., Grombacher T., Rarey M. (2010). Analyzing the topology of active sites: On the prediction of pockets and subpockets. J. Chem. Info. Model..

[16] Volkamer A., Kuhn D., Grombacher T., Rippmann F., Rarey M. (2012). Combining global and local measures for structure-based druggability predictions. J. Chem. Inf. Model..

[17] Mendez D., Gaulton A., Bento A.P., Chambers J., de Veij M., Félix E., Magariños M.P., Mosquera J.F., Mutowo P., Nowotka M. (2019). ChEMBL: Towards direct deposition of bioassay data. Nucleic Acids Res..

[18] Ehrman T.M., Barlow D.J., Hylands P.J. (2010). In silico search for multi-target anti-inflammatories in Chinese herbs and formulas. Bioorg. Med. Chem..

[19] Ju Z., Su M., Hong J., La Kim E., Moon H.R., Chung H.Y., Jung J.H. (2019). Design of balanced COX inhibitors based on anti-inflammatory and/or COX-2 inhibitory ascidian metabolites. Eur. J. Med. Chem..

[20] Eren G., Ünlü S., Nuñez M.T., Labeaga L., Ledo F., Entrena A., Banoglu E., Constantino G., Şahin M.F. (2010). Synthesis, biological evaluation, and docking studies of novel heterocyclic diaryl compounds as selective COX-2 inhibitors. Bioorg. Med. Chem..

[21] Yamamoto Y., Hisa T., Arai J., Saito Y., Yamamoto F., Mukai T., Ohshima T., Maeda M., Ohkubo Y. (2015). Isomeric methoxy analogs of nimesulide for development of brain cyclooxygense-2 (COX-2)-targeted imaging agents: Synthesis, in vitro COX-2-inhibitory potency, and cellular transport properties. Bioorg. Med. Chem..

[22] Beno B.R., Yeung K.S., Bartberger M.D., Pennington L.D., Meanwell N.A. (2015). A survey of the role of noncovalent sulfur interactions in drug design. J. Med. Chem..

[23] Berman H.M., Westbrook Z.F., Gilliland G., Bhat T.N., Weissing H., Shindyalov I.N., Bourne P.E. (2000). The Protein Data Bank. Nucleic Acids Res..

[24] Goujon M., McWilliam H., Li W., Valentin F., Squizzato S., Paern J., Lopez R. (2010). A new bioinformatics analysis tools framework at EMBL–EBI. Nucleic Acids Res..

[25] Martin F.J., Amode M.R., Aneja A., Austine-Orimoloye O., Azov A.G., Barnes I., Becker A., Bennett R., Berry A., Bhai J. (2023). Ensembl 2023. Nucleic Acids Res..

[26] Meyder A., Nittinger E., Lange G., Klein R., Rarey M. (2017). Estimating Electron Density Support for Individual Atoms and Molecular Fragments in X-ray Structure. J. Chem. Inf. Model..

[27] Schöning-Stierand K., Diedrich K., Ehrt C., Flachsenberg F., Graef J., Sieg J., Rarey M. (2022). Proteins Plus: A comprehensive collection of web-based molecular modeling tools. Nucleic Acids Res..

[28] Reddy M.R., Billa V.K., Pallela V.R., Mallireddigari M.R., Boominathan R., Gabriel J.L., Reddy E.P. (2008). Design, synthesis, and biological evaluation of 1-(4-sulfamylphenyl)-3-trifluoromethyl-5-indolyl pyrazolines as cyclooxygenase-2 (COX-2) and lipoxygenase (LOX) inhibitors. Bioorg. Med. Chem..

[29] Zarghi A., Zebardast T., Daraie B., Hedayati M. (2009). Design and synthesis of new 1, 3-benzthiazinan-4-one derivatives as selective cyclooxygenase (COX-2) inhibitors. Bioorg. Med. Chem..

[30] Shrivastava S.K., Srivastava P., Bandresh R., Tripathi P.N., Tripathi A. (2017). Design, synthesis, and biological evaluation of some novel indolizine derivatives as dual cyclooxygenase and lipoxygenase inhibitor for anti-inflammatory activity. Bioorg. Med. Chem..

[31] Magda A.A., Abdel-Aziz N.I., Alaa A.M., El-Azab A.S., ElTahir K.E. (2012). Synthesis, biological evaluation and molecular modeling study of pyrazole and pyrazoline derivatives as selective COX-2 inhibitors and anti-inflammatory agents. Part 2. Bioorg. Med. Chem..

[32] Kaur J., Bhardwaj A., Huang Z., Knaus E.E. (2012). N-1 and C-3 substituted indole Schiff bases as selective COX-2 inhibitors: Synthesis and biological evaluation. Bioorg. Med. Chem. Lett..

[33] Pérez D.J., Díaz-Reval M.I., Obledo-Benicio F., Zakai U.I., Gómez-Sandoval Z., Razo-Hernández R.S., West R., Sumaya-Martínez M.T., Pineda-Urbina K., Ramos-Organillo Á. (2017). Silicon containing ibuprofen derivatives with antioxidant and anti-inflammatory activities: An in vivo and in silico study. Eur. J. Pharmacol..

[34] Rodríguez-Lozada J., Tovar-Gudiño E., Guevara-Salazar J.A., Razo-Hernández R.S., Santiago Á., Pastor N., Fernández-Zertuche M. (2018). QSAR and molecular docking studies of the inhibitory activity of novel heterocyclic GABA analogues over GABA-AT. Molecules.

[35] Hehre W.J. (2003). A Guide to Molecular Mechanics and Quantum Chemical Calculations.

[36] Thomsen R., Christensen M.H. (2006). MolDock: A new technique for high-accuracy molecular docking. J. Med. Chem..

[37] Selinsky B.S., Gupta K., Sharkey C.T., Loll P.J. (2001). Structural analysis of NSAID binding by prostaglandin H2 synthase: Time-dependent and time-independent inhibitors elicit identical enzyme conformations. Biochemistry.

[38] Harman C.A., Turman M.V., Kozak K.R., Marnett L.J., Smith W.L., Garavito R.M. (2007). Structural basis of enantioselective inhibition of cyclooxygenase-1 by S-α-substituted Indomethacin ethanolamide. J. Biol. Chem..

[39] Rimon G., Sidhu R.S., Lauver D.A., Lee J.Y., Sharma N.P., Yuan C., Frieler R.A., Trievel R.C., Lucchesi B.R., Smith W.L. (2010). Coxibs interfere with the action of aspirin by binding tightly to one monomer of cyclooxygenase-1. Proc. Natl. Acad. Sci. USA.

[40] Xu S., Hermanson D.J., Banerjee S., Ghebreselasie K., Clayton G.M., Garavito R.M., Marnett L.J. (2014). Oxicams bind in a novel mode to the cyclooxygenase active site via a two-water-mediated H-bonding network. J. Biol. Chem..

[41] Orlando B.J., Lucido M.J., Malkowski M.G. (2015). The structure of ibuprofen bound to cyclooxygenase-2. J. Struct. Biol..

[42] Kurumbail R.G., Stevens A.M., Gierse J.K., McDonald J.J., Stegeman R.A., Pak J.Y., Gildehaus D., Iyashiro J.M., Penning T.D., Seibert K. (1996). Structural basis for selective inhibition of cyclooxygenase-2 by anti-inflammatory agents. Nature.

[43] Ferraroni M., Matera I., Steimer L., Bürger S., Scozzafava A., Stolz A., Briganti F. (2012). Crystal structures of salicylate 1, 2-dioxygenase-substrates adducts: A step towards the comprehension of the structural basis for substrate selection in class III ring cleaving dioxygenases. J. Struct. Biol..

