# Supplementary material for: A Computational Method for the Binding Mode Prediction of COX-1 and COX-2 Inhibitors: Analyzing the Union of Coxibs, Oxicams, Propionic and Acetic Acids
Source: Pharmaceuticals (Basel). 2023 Dec 4;16(12):1688. doi: 10.3390/ph16121688 (PMC10747940; doi:10.3390/ph16121688)
Supplement: Supplementary file 1 [file pharmaceuticals-16-01688-s001.zip › pharmaceuticals-2675373-supplementary.pdf]

Supporting Information

For

A computational method for the binding mode prediction of COX-1 and COX-2 inhibitors: analyzing the union of coxibs, oxicams, propionic and acetic acids.

Estefany Bello -Vargas [1], Mario Alberto Leyva-Peralta [2], Zeferino Gómez-Sandoval [3], Mario Ordóñez[1], Rodrigo Said Razo-Hernández[4].

Secuencial analysis

```
MOUSE_COX1------MSRRSLSLWFPLLLLLLLPPTPSVLLADPGVPSPVNPCCYYPCQNQGVCVRFGLDNYQCD 60
6Y3C_1------------------------------------ADPGAPTPVNPCCYYPCQHQGICVRFGLDRYQCD 34
1EQG_1--------------------------------PVFSADPGAPAPVNPCCYYPCQHQGICVRFGLDRYQCD 38
2OYU_1----------MSRQSISLRFPLLLLLLSP--SPVFSADPGAPAPVNPCCYYPCQHQGICVRFGLDRYQCD 58
3KK6_1-------------------------------------------PVNPCCYYPCQHQGICVRFGLDRYQCD 27
4O1Z_1-------------------------------------------PVNPCCYYPCQHQGICVRFGLDRYQCD 27
                                                 ***********:**:*******.****

MOUSE_COX1------CTRTGYSGPNCTIPEIWTWLRNSLRPSPSFTHFLLTHGYWLWEFVNATFIREVLMRLVLT 120
6Y3C_1----------CTRTGYSGPNCTIPGLWTWLRNSLRPSPSFTHFLLTHGRWFWEFVNATFIREMLMRLVLT 94
1EQG_1----------CTRTGYSGPNCTIPEIWTWLRTTLRPSPSFIHFLLTHGRWLWDFVNATFIRDTLMRLVLT 98
2OYU_1----------CTRTGYSGPNCTIPEIWTWLRTTLRPSPSFIHFLLTHGRWLWDFVNATFIRDTLMRLVLT 118
3KK6_1----------CTRTGYSGPNCTIPEIWTWLRTTLRPSPSFIHFLLTHGRWLWDFVNATFIRDTLMRLVLT 87
4O1Z_1----------CTRTGYSGPNCTIPEIWTWLRTTLRPSPSFIHFLLTHGRWLWDFVNATFIRDTLMRLVLT 87
                **************  :*****.:******* ******* *:*:********: *******

MOUSE_COX1------VRSNLIPSPPTYNSAHDYISWESFSNVSYYTRILPSVPKDCPTPMGTKGKKQLPDVQLLA 180
6Y3C_1----------VRSNLIPSPPTYNSAHDYISWESFSNVSYYTRILPSVPKDCPTPMGTKGKKQLPDAQLLA 154
1EQG_1----------VRSNLIPSPPTYNIAHDYISWESFSNVSYYTRILPSVPRDCPTPMGTKGKKQLPDAEFLS 158
2OYU_1----------VRSNLIPSPPTYNIAHDYISWESFSNVSYYTRILPSVPRDCPTPMGTKGKKQLPDAEFLS 178
3KK6_1----------VRSNLIPSPPTYNIAHDYISWESFSNVSYYTRILPSVPRDCPTPMGTKGKKQLPDAEFLS 147
4O1Z_1----------VRSNLIPSPPTYNIAHDYISWESFSNVSYYTRILPSVPRDCPTPMGTKGKKQLPDAEFLS 147
                ************* ************************:****************.::*:

MOUSE_COX1------QQLLLRREFIPAPQGTNILFAFFAQHFTHQFFKTSGKMGPGFTKALGHGVDLGHIYGDNL 240
6Y3C_1----------RRFLLRRKFIPDPQGTNLMFAFFAQHFTHQFFKTSGKMGPGFTKALGHGVDLGHIYGDNL 214
1EQG_1----------RRFLLRRKFIPDPQGTNLMFAFFAQHFTHQFFKTSGKMGPGFTKALGHGVDLGHIYGDNL 218
2OYU_1----------RRFLLRRKFIPDPQGTNLMFAFFAQHFTHQFFKTSGKMGPGFTKALGHGVDLGHIYGDNL 238
3KK6_1----------RRFLLRRKFIPDPQGTNLMFAFFAQHFTHQFFKTSGKMGPGFTKALGHGVDLGHIYGDNL 207
4O1Z_1----------RRFLLRRKFIPDPQGTNLMFAFFAQHFTHQFFKTSGKMGPGFTKALGHGVDLGHIYGDNL 207
                :::****:*** *****::*****************************************

MOUSE_COX1------ERQYHLRLFKDGKLKYQVLDGEVYPPSVEQASVLMRYPPGVPPERQMAVGQEVFGLLPGL 300
6Y3C_1----------ERQYQLRLFKDGKLKYQVLDGEMYPPSVEEAPVLMHYPRGIPPQSQMAVGQEVFGLLPGL 274
1EQG_1----------ERQYQLRLFKDGKLKYQMLNGEVYPPSVEEAPVLMHYPRGIPPQSQMAVGQEVFGLLPGL 278
2OYU_1----------ERQYQLRLFKDGKLKYQMLNGEVYPPSVEEAPVLMHYPRGIPPQSQMAVGQEVFGLLPGL 298
3KK6_1----------ERQYQLRLFKDGKLKYQMLNGEVYPPSVEEAPVLMHYPRGIPPQSQMAVGQEVFGLLPGL 267
4O1Z_1----------ERQYQLRLFKDGKLKYQMLNGEVYPPSVEEAPVLMHYPRGIPPQSQMAVGQEVFGLLPGL 267
                ****:************:*:**:******:* ***:** *:**: ***************

MOUSE_COX1------MLFSTIWLREHNRVCDLLKEEHPTWDDEQLFQTTRLILIGETIKIVIEEYVQHLSGYFLQ 360
6Y3C_1----------MLYATLWLREHNRVCDLLKAEHPTWGDEQLFQTTRLILIGETIKIVIEEYVQQLSGYFLQ 334
1EQG_1----------MLYATIWLREHNRVCDLLKAEHPTWGDEQLFQTARLILIGETIKIVIEEYVQQLSGYFLQ 338
2OYU_1----------MLYATIWLREHNRVCDLLKAEHPTWGDEQLFQTARLILIGETIKIVIEEYVQQLSGYFLQ 358
3KK6_1----------MLYATIWLREHNRVCDLLKAEHPTWGDEQLFQTARLILIGETIKIVIEEYVQQLSGYFLQ 327
4O1Z_1----------MLYATIWLREHNRVCDLLKAEHPTWGDEQLFQTARLILIGETIKIVIEEYVQQLSGYFLQ 327
                **::*:************* *****.*******:******************:*******

MOUSE_COX1------LKFDPELLFRAQFQYRNRIAMEFNHLYHWHPLMPNSFQVGSQEYSYEQFLFNTSMLVDYG 420
6Y3C_1----------LKFDPELLFGVQFQYRNRIAMEFNHLYHWHPLMPDSFKVGSQEYSYEQFLFNTSMLVDYG 394
1EQG_1----------LKFDPELLFGAQFQYRNRIAMEFNQLYHWHPLMPDSFRVGPQDYSYEQFLFNTSMLVDYG 398
2OYU_1----------LKFDPELLFGAQFQYRNRIAMEFNQLYHWHPLMPDSFRVGPQDYSYEQFLFNTSMLVDYG 418
3KK6_1----------LKFDPELLFGAQFQYRNRIAMEFNQLYHWHPLMPDSFRVGPQDYSYEQFLFNTSMLVDYG 387
4O1Z_1----------LKFDPELLFGAQFQYRNRIAMEFNQLYHWHPLMPDSFRVGPQDYSYEQFLFNTSMLVDYG 387
```

```
                *********  .*************:*********:**:** *:*****************
MOUSE_COX1------VEALVDAFSRQRAGRIGGGRNFDYHVLHVAVDVIKESREMRLQPFNEYRKRFGLKPYTSF 480
6Y3C_1----------VEALVDAFSRQIAGRIGGGRNMDHHILHVAVDVIRESREMRLQPFNEYRKRFGMKPYTSF 454
1EQG_1----------VEALVDAFSRQPAGRIGGGRNIDHHILHVAVDVIKESRVLRLQPFNEYRKRFGMKPYTSF 458
2OYU_1----------VEALVDAFSRQPAGRIGGGRNIDHHILHVAVDVIKESRVLRLQPFNEYRKRFGMKPYTSF 478
3KK6_1----------VEALVDAFSRQPAGRIGGGRNIDHHILHVAVDVIKESRVLRLQPFNEYRKRFGMKPYTSF 447
4O1Z_1----------VEALVDAFSRQPAGRIGGGRNIDHHILHVAVDVIKESRVLRLQPFNEYRKRFGMKPYTSF 447
                *********** *********:*:*:********:*** :*************:******
MOUSE_COX1------QELTGEKEMAAELEELYGDIDALEFYPGLLLEKCQPNSIFGESMIEMGAPFSLKGLLGNP 540
6Y3C_1----------QELVGEKEMAAELEELYGDIDALEFYPGLLLEKCHPNSIFGESMIEIGAPFSLKGLLGNP 514
1EQG_1----------QELTGEKEMAAELEELYGDIDALEFYPGLLLEKCHPNSIFGESMIEMGAPFSLKGLLGNP 518
2OYU_1----------QELTGEKEMAAELEELYGDIDALEFYPGLLLEKCHPNSIFGESMIEMGAPFSLKGLLGNP 538
3KK6_1----------QELTGEKEMAAELEELYGDIDALEFYPGLLLEKCHPNSIFGESMIEMGAPFSLKGLLGNP 507
4O1Z_1----------QELTGEKEMAAELEELYGDIDALEFYPGLLLEKCHPNSIFGESMIEMGAPFSLKGLLGNP 507
                ***.******************************:***********:*************


MOUSE_COX1------ICSPEYWKPSTFGGDVGFNLVNTASLKKLVCLNTKTCPYVSFRVPDYPGDDGSVLVRRST 600
6Y3C_1----------ICSPEYWKPSTFGGEVGFNIVKTATLKKLVCLNTKTCPYVSFRVPDASQDDGPAVERPST 574
1EQG_1----------ICSPEYWKASTFGGEVGFNLVKTATLKKLVCLNTKTCPYVSFHVPDPRQEDRPGVERPPT 578
2OYU_1----------ICSPEYWKASTFGGEVGFNLVKTATLKKLVCLNTKTCPYVSFHVPDPRQEDRPGVERPPT 598
3KK6_1----------ICSPEYWKASTFGGEVGFNLVKTATLKKLVCLNTKTCPYVSFHVPD-------------- 553
4O1Z_1----------ICSPEYWKASTFGGEVGFNLVKTATLKKLVCLNTKTCPYVSFHVPDPRQEDRPGVERPPT 567
                ******** *****:****:*:**:*****************:***
MOUSE_COX1_SEQUENCE      EL       602
6Y3C_1|Chain             EL       576
1EQG_1|Chains            EL       580
2OYU_1|Chain             EL       600
3KK6_1|Chains            --       553
4O1Z_1|Chains            EL       569
```

Figure S1. Comparative sequence analysis for COX-1. The aminoacid residues inside box line black

```
Human_cox2------MLARALLLCAVLALSHTANPCCSHPCQNRGVCMSVGFDQYKCDCTRTGFYGENCSTPEFL 60
SHEEP_COX2------MLARALLLCAAVALCGAANPCCSHPCQNRGVCMSVGFDQYKCDCTRTGFYGENCTTPEFL 60
4PH9_1---------------------------HHPCCSNPCQNRGECMSTGFDQYKCDCTRTGFYGENCTTPEFL 43
4COX_1---------------------------ANPCCSNPCQNRGECMSTGFDQYKCDCTRTGFYGENCTTPEFL 43
3LN1_1---------------------------ANPCCSNPCQNRGECMSTGFDQYKCDCTRTGFYGENCTTPEFL 43
4M11_1---------------------------ANPCCSNPCQNRGECMSTGFDQYKCDCTRTGFYGENCTTPEFL 43
                                  :****:****** ***.********************:*****
Human_cox2------TRIKLFLKPTPNTVHYILTHFKGFWNVVNNIPFLRNAIMSYVLTSRSHLIDSPPTYNADY 120
SHEEP_COX2------TRIKLLLKPTPDTVHYILTHFKGVWNIVNKISFLRNMIMRYVLTSRSHLIESPPTYNVHY 120
4PH9_1----------TRIKLLLKPTPNTVHYILTHFKGVWNIVNNIPFLRSLIMKYVLTSRSYLIDSPPTYNVHY 103
4COX_1----------TRIKLLLKPTPNTVHYILTHFKGVWNIVNNIPFLRSLIMKYVLTSRSYLIDSPPTYNVHY 103
3LN1_1----------TRIKLLLKPTPNTVHYILTHFKGVWNIVNNIPFLRSLIMKYVLTSRSYLIDSPPTYNVHY 103
4M11_1----------TRIKLLLKPTPNTVHYILTHFKGVWNIVNNIPFLRSLIMKYVLTSRSYLIDSPPTYNVHY 103
                *****:*****:***********.**:**:* ***. ** *******:**:******..*
Human_cox2------GYKSWEAFSNLSYYTRALPPVPDDCPTPLGVKGKKQLPDSNEIVEKLLLRRKFIPDPQGS 180
SHEEP_COX2------SYKSWEAFSNLSYYTRALPPVPDDCPTPMGVKGRKELPDSKEVVKKVLLRRKFIPDPQGT 180
4PH9_1----------GYKSWEAFSNLSYYTRALPPVADDCPTPMGVKGNKELPDSKEVLEKVLLRREFIPDPQGS 163
4COX_1----------GYKSWEAFSNLSYYTRALPPVADDCPTPMGVKGNKELPDSKEVLEKVLLRREFIPDPQGS 163
3LN1_1----------GYKSWEAFSNLSYYTRALPPVADDCPTPMGVKGNKELPDSKEVLEKVLLRREFIPDPQGS 163
4M11_1----------GYKSWEAFSNLSYYTRALPPVADDCPTPMGVKGNKELPDSKEVLEKVLLRREFIPDPQGS 163
                .******************** ******:****.*:****:*:::*:****:*******:
Human_cox2-----NMMFAFFAQHFTHQFFKTDHKRGPAFTNGLGHGVDLNHIYGETLARQRKLRLFKDGKMKY  240
SHEEP_COX2------NLMFAFFAQHFTHQFFKTDIERGPAFTKGKNHGVDLSHVYGESLERQHKLRLFKDGKMKY 240
4PH9_1----------NMMFAFFAQHFTHQFFKTDHKRGPGFTRGLGHGVDLNHIYGETLDRQHKLRLFKDGKLKY 223
4COX_1----------NMMFAFFAQHFTHQFFKTDHKRGPGFTRGLGHGVDLNHIYGETLDRQHKLRLFKDGKLKY 223
3LN1_1----------NMMFAFFAQHFTHQFFKTDHKRGPGFTRGLGHGVDLNHIYGETLDRQHKLRLFKDGKLKY 223
4M11_1----------NMMFAFFAQHFTHQFFKTDHKRGPGFTRGLGHGVDLNHIYGETLDRQHKLRLFKDGKLKY 223
                *:***************** :***.**.* .*****.*:***:* **:*********:**
Human_cox2------QIIDGEMYPPTVKDTQAEMIYPPQVPEHLRFAVGQEVFGLVPGLMMYATIWLREHNRVCD 300
SHEEP_COX2------QMINGEMYPPTVKDTQVEMIYPPHIPEHLKFAVGQEVFGLVPGLMMYATIWLREHNRVCD 300
4PH9_1----------QVIGGEVYPPTVKDTQVEMIYPPHIPENLQFAVGQEVFGLVPGLMMYATIWLREHNRVCD 283
```

```
4COX_1----------QVIGGEVYPPTVKDTQVEMIYPPHIPENLQFAVGQEVFGLVPGLMMYATIWLREHQRVCD 283
3LN1_1----------QVIGGEVYPPTVKDTQVEMIYPPHIPENLQFAVGQEVFGLVPGLMMYATIWLREHNRVCD 283
4M11_1----------QVIGGEVYPPTVKDTQVEMIYPPHIPENLQFAVGQEVFGLVPGLMMYATIWLREHNRVCD 283
                *:*.**:*********.******::**:*:*************************:****
Human_cox2------VLKQEHPEWGDEQLFQTSRLILIGETIKIVIEDYVQHLSGYHFKLKFDPELLFNKQFQYQ 360
SHEEP_COX2------VLKQEHPEWGDEQLFQTSRLILIGETIKIVIEDYVQHLSGYHFKLKFDPELLFNQQFQYQ 360
4PH9_1----------ILKQEHPEWGDEQLFQTSRLILIGETIKIVIEDYVQHLSGYHFKLKFDPELLFNQQFQYQ 343
4COX_1----------ILKQEHPEWGDEQLFQTSKLILIGETIKIVIEDYVQHLSGYHFKLKFDPELLFNQQFQYQ 343
3LN1_1----------ILKQEHPEWGDEQLFQTSRLILIGETIKIVIEDYVQHLSGYHFKLKFDPELLFNQQFQYQ 343
4M11_1----------ILKQEHPEWGDEQLFQTSRLILIGETIKIVIEDYVQHLSGYHFKLKFDPELLFNQQFQYQ 343
                :*****************:***********************************:*****
Human_cox2------NRIAAEFNTLYHWHPLLPDTFQIHDQKYNYQQFIYNNSILLEHGITQFVESFTRQIAGRV 420
SHEEP_COX2------NRIAAEFNTLYHWHPLLPDVFQIDGQEYNYQQFIYNNSVLLEHGVTQFVESFTRQIAGRV 420
4PH9_1----------NRIASEFNTLYHWHPLLPDTFNIEDQEYSFKQFLYNNSILLEHGLTQFVESFTRQIAGRV 403
4COX_1----------NRIASEFNTLYHWHPLLPDTFNIEDQEYSFKQFLYNNSILLEHGLTQFVESFTRQIAGRV 403
3LN1_1----------NRIASEFNTLYHWHPLLPDTFNIEDQEYSFKQFLYNNSILLEHGLTQFVESFTRQIAGRV 403
4M11_1----------NRIASEFNTLYHWHPLLPDTFNIEDQEYSFKQFLYNNSILLEHGLTQFVESFTRQIAGRV 403
                ****:**************.*:*..*:*.::**:****:*****:***************

Human_cox2------AGGRNVPPAVQKVSQASIDQSRQMKYQSFNEYRKRFMLKPYESFEELTGEKEMSAELEAL 480
SHEEP_COX2------AGGRNLPAAVEKVSKASLDQSREMKYQSFNEYRKRFLLKPYESFEELTGEKEMAAELEAL 480
4PH9_1----------AGGRNVPIAVQAVAKASIDQSREMKYQSLNEYRKRFSLKPYTSFEELTGEKEMAAELKAL 463
4COX_1----------AGGRNVPIAVQAVAKASIDQSREMKYQSLNEYRKRFSLKPYTSFEELTGEKEMAAELKAL 463
3LN1_1----------AGGRNVPIAVQAVAKASIDQSREMKYQSLNEYRKRFSLKPYTSFEELTGEKEMAAELKAL 463
4M11_1----------AGGRNVPIAVQAVAKASIDQSREMKYQSLNEYRKRFSLKPYTSFEELTGEKEMAAELKAL 463
                *****:* **: *::**:****:*****:******* **** ***********:***:**

Human_cox2------YGDIDAVELYPALLVEKPRPDAIFGETMVEVGAPFSLKGLMGNVICSPAYWKPSTFGGEV 540
SHEEP_COX2------YGDIDAMELYPALLVEKPRPDAIFGETMVEAGAPFSLKGLMGNPICSPEYWKPSTFGGEV 540
4PH9_1----------YSDIDVMELYPALLVEKPRPDAIFGETMVELGAPFSLKGLMGNPICSPQYWKPSTFGGEV 523
4COX_1----------YSDIDVMELYPALLVEKPRPDAIFGETMVELGAPFSLKGLMGNPICSPQYWKPSTFGGEV 523
3LN1_1----------YSDIDVMELYPALLVEKPRPDAIFGETMVELGAPFSLKGLMGNPICSPQYWKPSTFGGEV 523
4M11_1----------YSDIDVMELYPALLVEKPRPDAIFGETMVELGAPFSLKGLMGNPICSPQYWKPSTFGGEV 523
                *.***.:*********************** ************ **** ***********

Human_cox2------GFQIINTASIQSLICNNVKGCPFTSFSVPDPELIKTVTINASSSRSGLDDINPTVLLKER 600
SHEEP_COX2------GFKIINTASIQSLICSNVKGCPFTSFSVQDAHLTKTVTINASSSHSGLDDINPTVLLKER 600
4PH9_1----------GFKIINTASIQSLICNNVKGCPFTSFNV-------------------------------- 551
4COX_1----------GFKIINTASIQSLICNNVKGCPFTSFNVQDPQPTKTATINASASHSRLDDINPTVLIKRR 583
3LN1_1----------GFKIINTASIQSLICNNVKGCPFTSFNVQDPQPTKTATINASASHSRLDDINPTVLIKRR 583
4M11_1----------GFKIINTASIQSLICNNVKGCPFTSFNVQ------------------------------- 552
                **:************.**********.*

Human_cox2--------STEL    604
SHEEP_COX2--------STEL    604
4PH9_1----------------    551
4COX_1------------STEL    587
3LN1_1------------STEL    587
4M11_1----------------    552
```

Figure S2. Comparative sequence analysis for COX-2. The aminoacid residues inside box line black

Results docking molecular

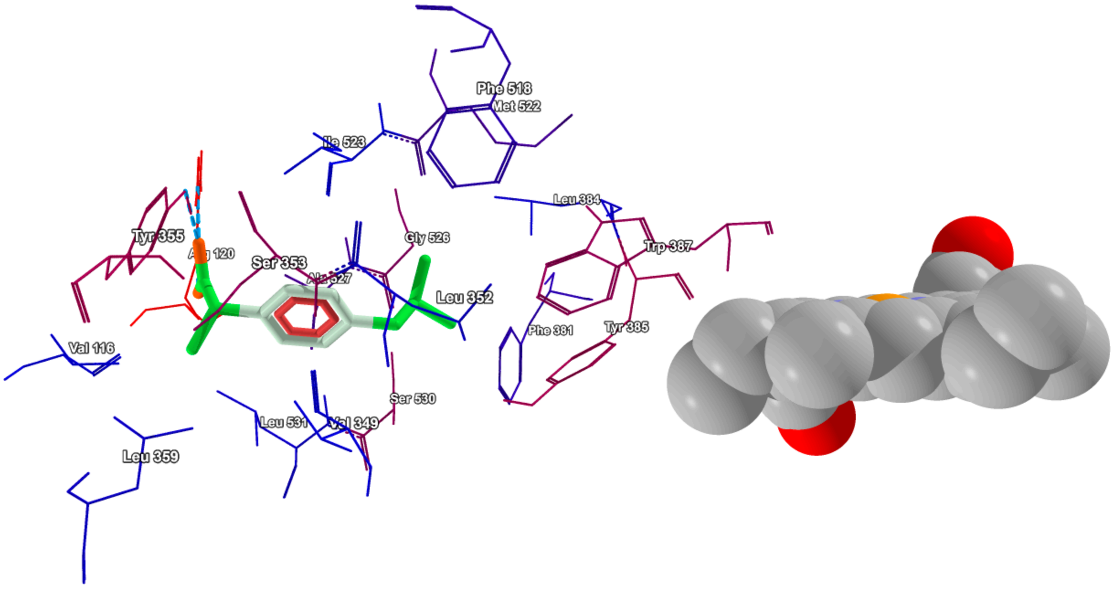

Figure S3. Binding mode of Ibuprofen in 1EQG crystal (COX-1).

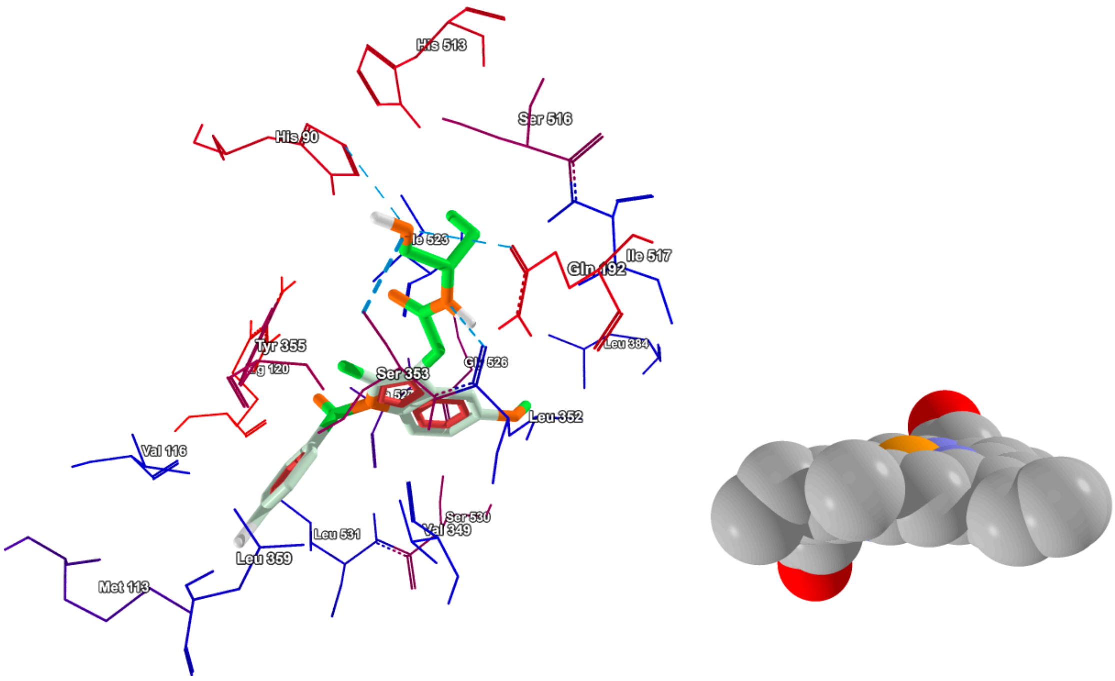

Figure S4. Binding mode of Ibuprofen in 2OYU crystal (COX-1).

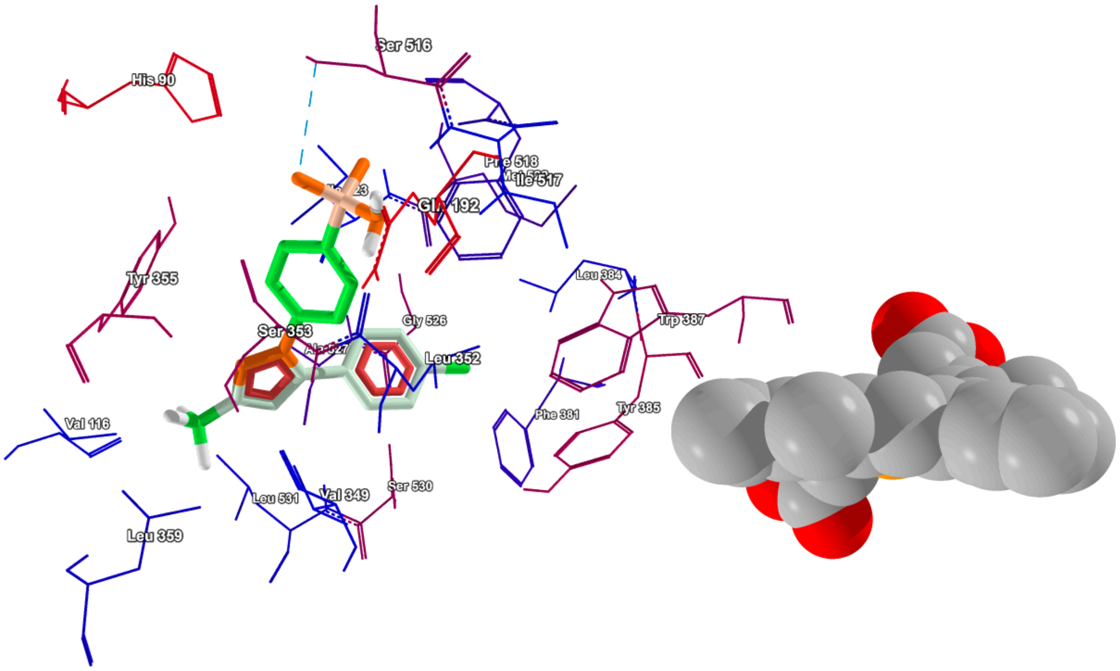

Figure S5. Binding mode of Ibuprofen in 3KK6 crystal (COX-1).

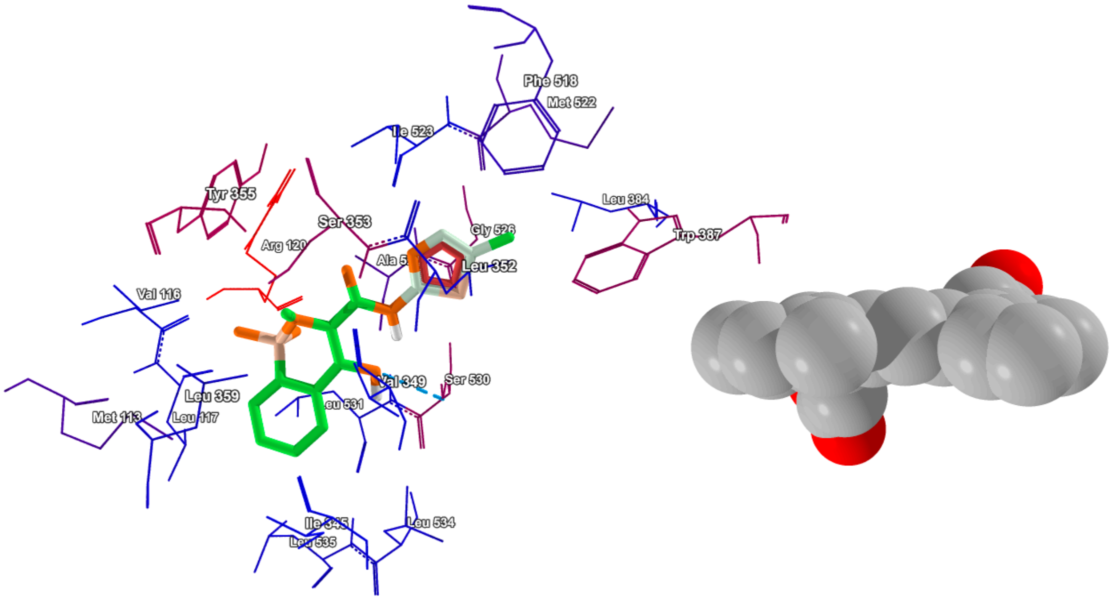

Figure S6. Binding mode of Ibuprofen in 4O1Z crystal (COX-1).

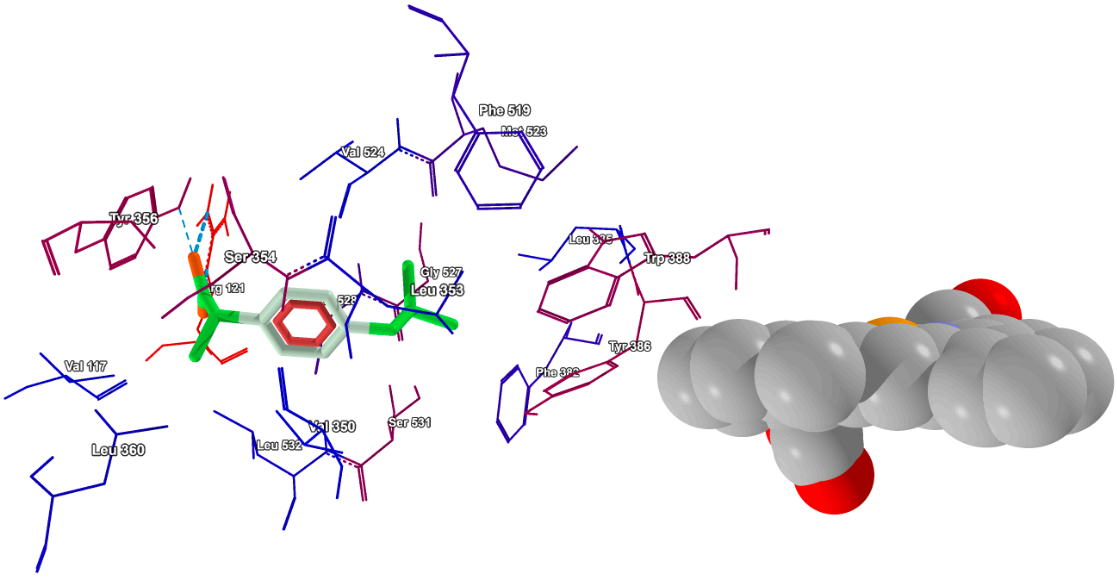

Figure S7. Binding mode of Ibuprofen in 4PH9 crystal (COX-2).

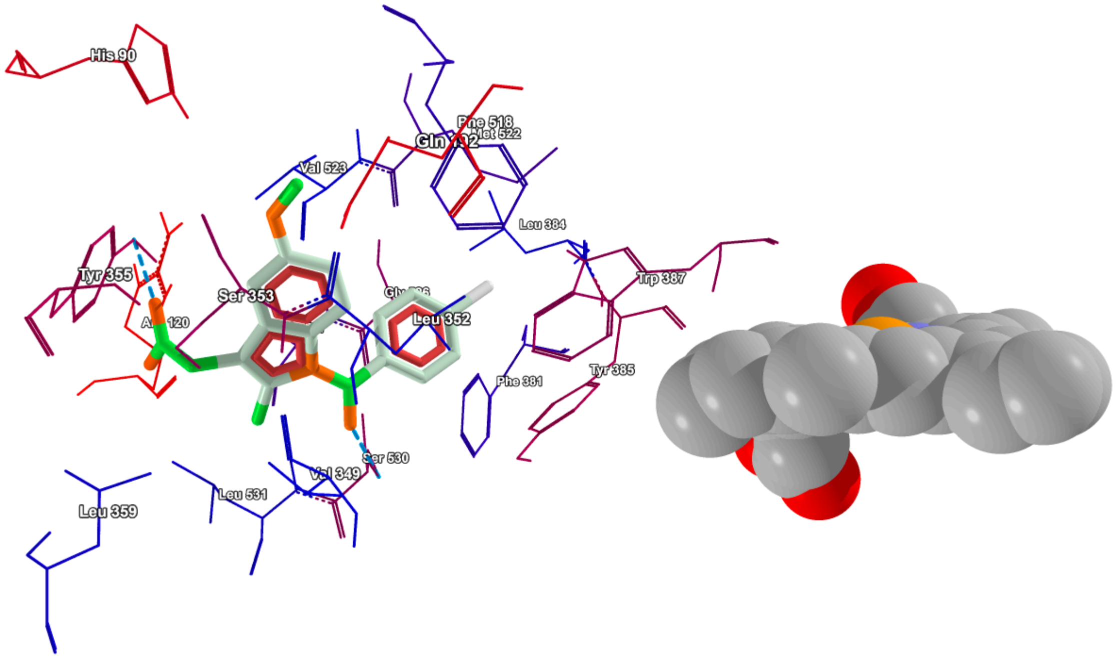

Figure S8. Binding mode of Indomethacin in 4COXcrystal (COX-2).

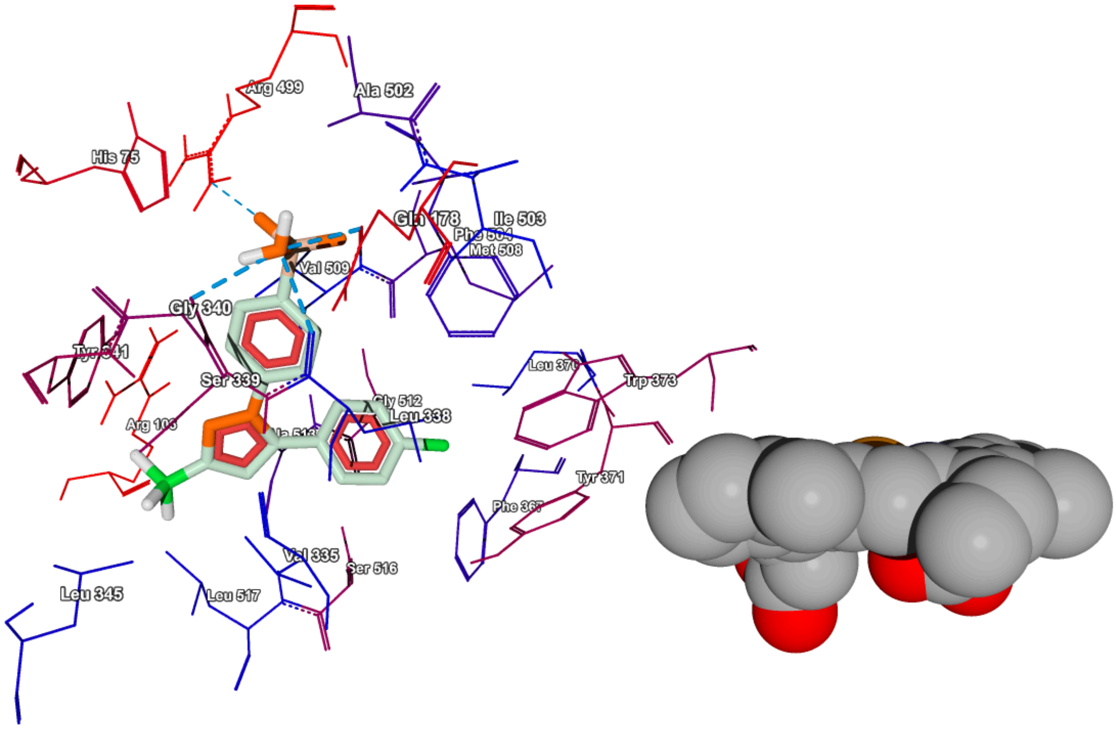

Figure S9. Binding mode of Celecoxib in 3LN1 crystal (COX-2).

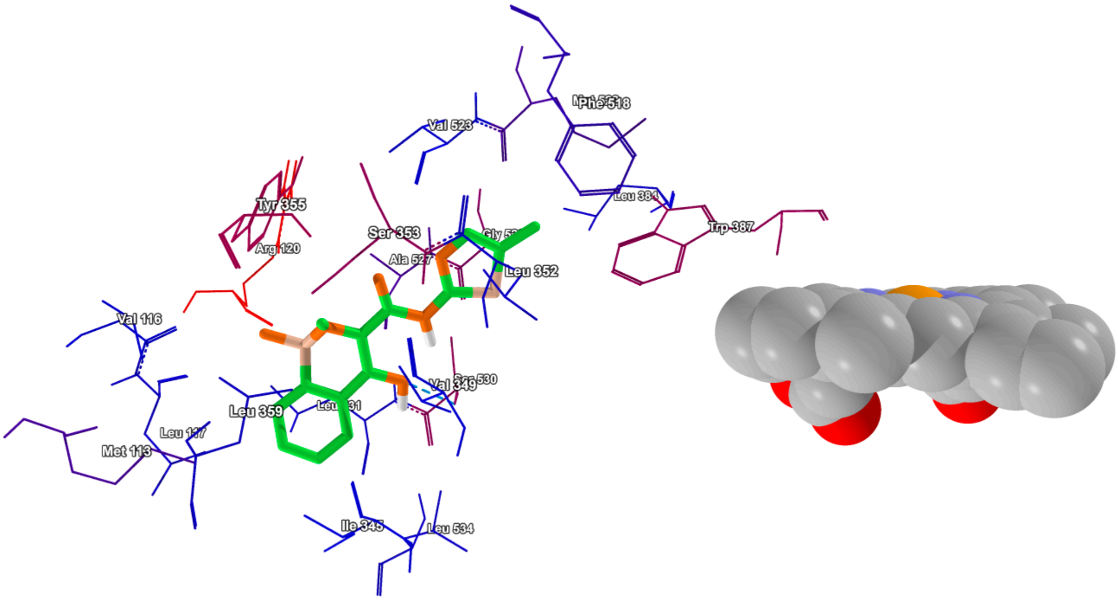

Figure S10. Binding mode of Meloxicam in 4M11 crystal (COX-2).

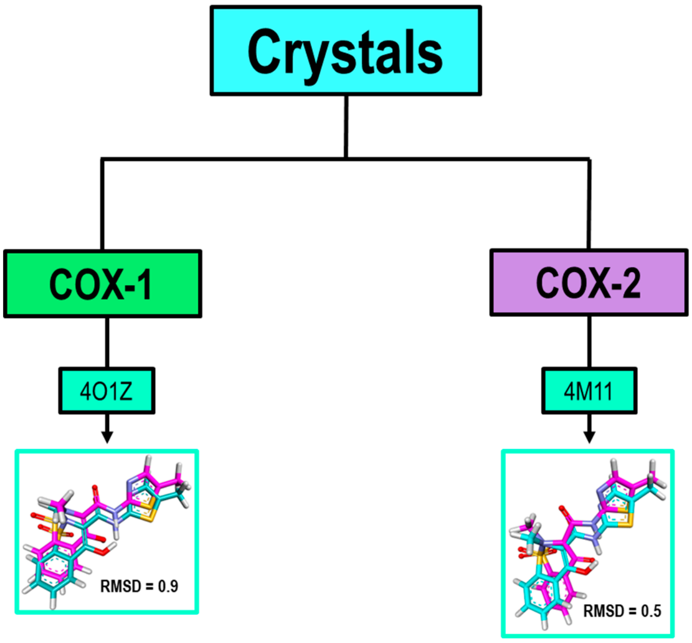

Figure S11. RMSD values obtained of structural validation of computational model with **Meloxicam** (Magenta) presented a parallel orientation of the nitrogen atom of the thiazole ring to the carbonyl of the amide original pose and color pose obtained in the molecule after experiment as show: Meloxicam (cyan).

Anti-inflammatory compound

Table S1. IC_{50} Values of NSAIDs and anti-inflammatory compounds.

**Compounds**	**IC_{50} values (μM)**		**Compounds**	**IC_{50} values (μM)**	
	**COX-1**	**COX-2**		**COX-1**	**COX-2**
MW: 244.27	0.59	3.1	MW: 263.13-	1.8	2.1
MW: 381.08	50	0.28	MW: 271.07	78.7 ± 3.60	0.3 ± 0.04
MW: 357.08	0.13	5.7	MW: 349.08	1.92 ± 0.9	8.21 ± 1.6

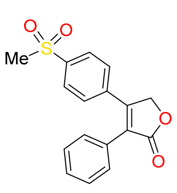 MW: 314.06	100	0.39	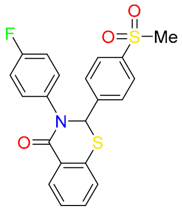 MW: 413.06	12.95	0.05
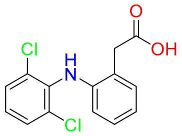 MW: 295.02	18.79	1.24	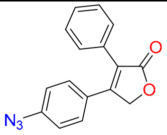 MW: 277.09	159.72	0.196
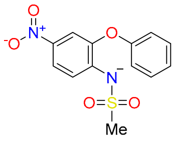 MW: 307.04	9.2	0.52	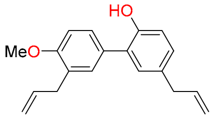 MW: 280.15	0.1	0.06
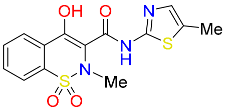 MW: 353.05	19.4	1.37	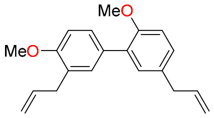 MW: 294.16	11.4	7.7
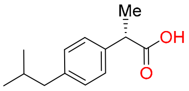 MW: 206.13	2.9	1.1	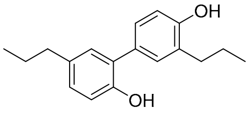 MW: 270.16	0.8	2.1
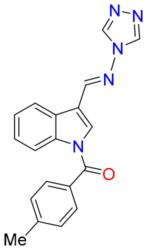 MW: 329.13 **30**	7.89	0.98	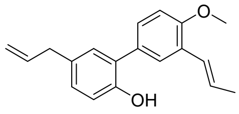 MW: 280.15	0.7	0.2
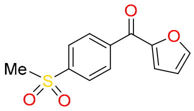 MW: 250.03	15.1 ± 0.94	19.2 ± 1.80	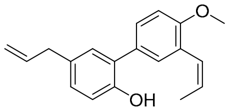 MW: 280.15	2.8	0.9
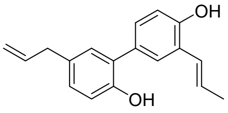 MW: 266.13	0.6	2.7			

Interaction energy values (Kcal/mol) of COXs inhibitors

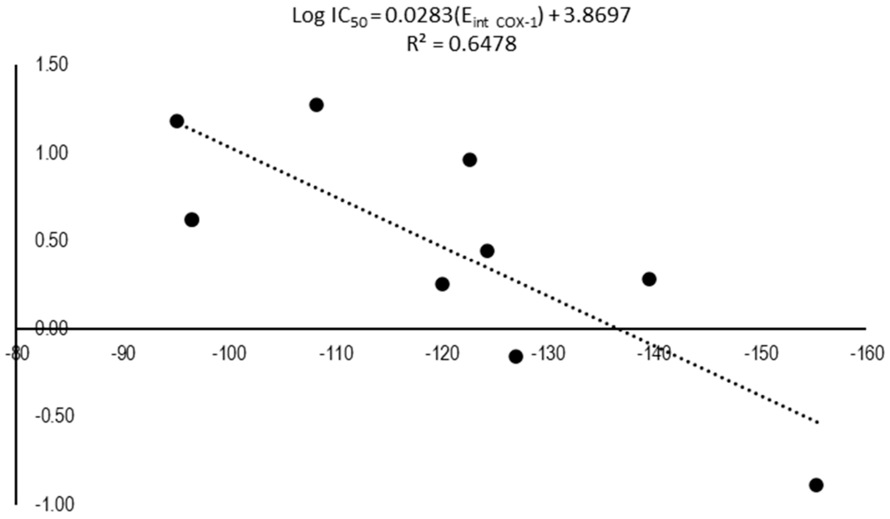

Figure S12.

Table S2. Interaction energy values (Kcal/mol) of COX-1 inhibitors that were coupled to the 1EQG crystal. Log $IC_{50\,exp}$, Log $IC_{50\,calc}$ and delta error (Δ error) values as show.

Compounds	Energy	Log $IC_{50\,exp}$	Log $IC_{50\,calc}$	Δ error
Ibuprofen[1]	-96.57	0.61	1.14	0.53
13	-120.03	0.26	0.47	0.21
19	-127.04	-0.15	0.27	0.12
20	-124.25	0.45	0.35	-0.1
29	-139.49	0.28	-0.08	-0.36
34	-95.09	1.18	1.18	0
Diclofenac	-108.23	1.27	0.81	-0.46
Indomethacine	-155.27	-0.89	-0.52	-1.41
Nimesulide	-122.59	0.96	0.40	-0.56

[1] Ligand corresponding to the crystal.

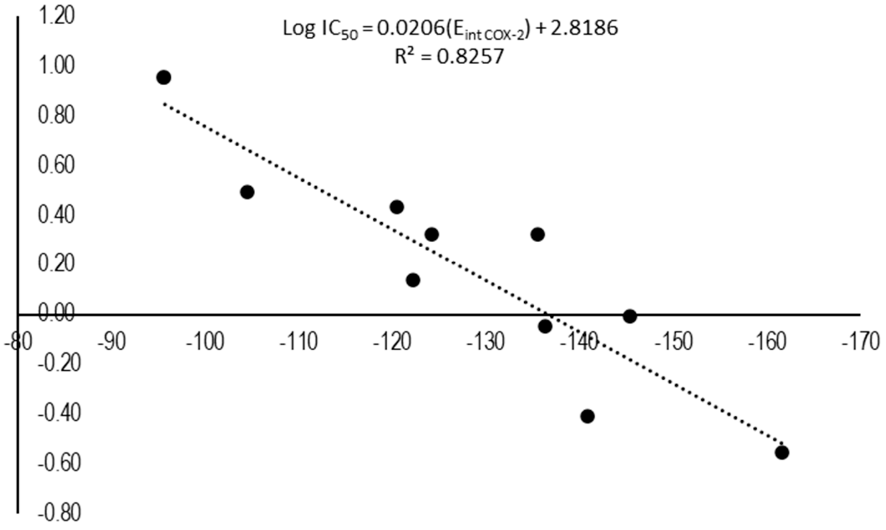

Figure S13.

Table S3. Interaction energy values (Kcal/mol) of COX-2 inhibitors that were coupled to the 4PH9 crystal. Log $IC_{50\ exp}$, Log $IC_{50\ calc}$ and delta error (Δ error) values as show.

Compounds	Energy	Log $IC_{50\ exp}$	Log $IC_{50\ calc}$	Δ error
Ibuprofen[1]	-95.66	0.95	0.85	-0.1
13	-135.53	0.32	0.03	-0.29
16	-124.25	0.32	0.26	-0.06
20	-136.32	-0.05	0.01	0.06
21	-120.5	0.43	0.34	-0.09
30	-145.4	-0.01	-0.18	-0.17
Celecoxib	-161.57	-0.55	-0.51	0.04
Flurbiprofen	-104.45	0.49	0.67	0.18
Meloxicam	-122.27	0.14	0.30	0.16

[1] Ligand corresponding to the crystal.

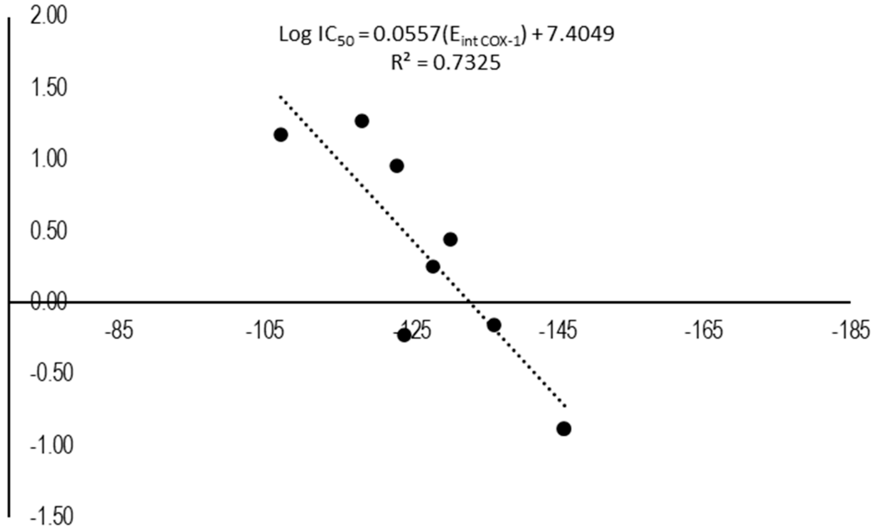

Figure S14.

Table S4. Interaction energy values (Kcal/mol) of COX-1 inhibitors that were coupled to the 2OYU crystal. Log $IC_{50\ exp}$, Log $IC_{50\ calc}$ and delta error (Δerror) values as show.

Compounds	Energy	Log $IC_{50\ exp}$	Log $IC_{50\ calc}$	Δ error
Indomethacin[1]	-145.91	-0.89	-0.72	0.17
13	-127.84	0.26	0.28	0.02
19	-136.29	-0.15	-0.19	-0.04
20	-130.2	0.45	0.15	-0.3
21	-123.93	-0.22	0.50	0.72
34	-107.02	1.18	1.44	0.26
Diclofenac	-118.2	1.27	0.82	-0.45
Nimesulide	-122.99	0.96	0.55	-0.41

[1] Ligand corresponding to the crystal.

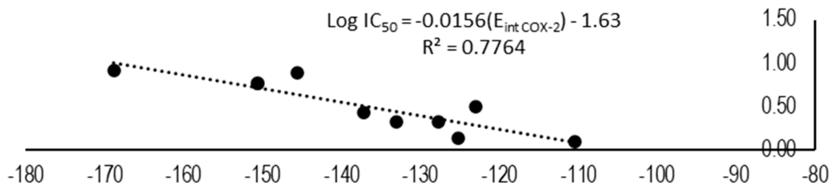

Figure S15.

Table S5. Interaction energy values (Kcal/mol) of COX-2 inhibitors that were coupled to the 4COX crystal. Log $IC_{50\ exp}$, Log $IC_{50\ calc}$ and delta error (Δ error) values as show.

Compounds	Energy	Log $IC_{50\ exp}$	Log $IC_{50\ calc}$	Δ error
Indomethacin[1]	-150.6	0.76	0.72	-0.04
13	-127.7	2.1	2.0	-0.1
15	-145.69	0.89	0.64	-0.25
16	-133.07	0.32	0.45	0.13
21	-137.15	0.43	0.51	0.08
29	-168.87	0.91	1.00	0.09
Diclofenac	-110.47	0.09	0.09	0
Flurbiprofen	-123.05	0.49	0.29	-0.2

[1] Ligand corresponding to the crystal.

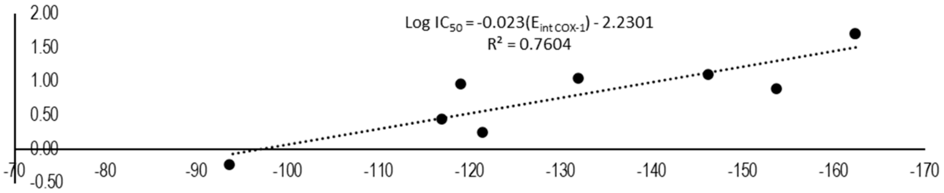

Figure S16.

Table S6. Interaction energy values (Kcal/mol) of COX-1 inhibitors that were coupled to the 3KK6 crystal. Log $IC_{50\ exp}$, Log $IC_{50\ calc}$ and delta error (Δ error) values as show.

Compounds	Energy	Log $IC_{50\ exp}$	Log $IC_{50\ calc}$	Δ error
Celecoxib[1]	-162.39	1.70	1.51	-0.19
13	-121.38	0.26	0.56	0.3
15	-131.88	1.06	0.80	-0.26
20	-116.91	0.45	0.46	0.01
26	-146.17	1.11	1.13	0.02
30	-153.73	0.90	1.31	0.41
Flurbiprofen	-93.58	-0.23	-0.08	0.15
Nimesulide	-119.00	0.96	0.51	-0.45

[1] Ligand corresponding to the crystal.

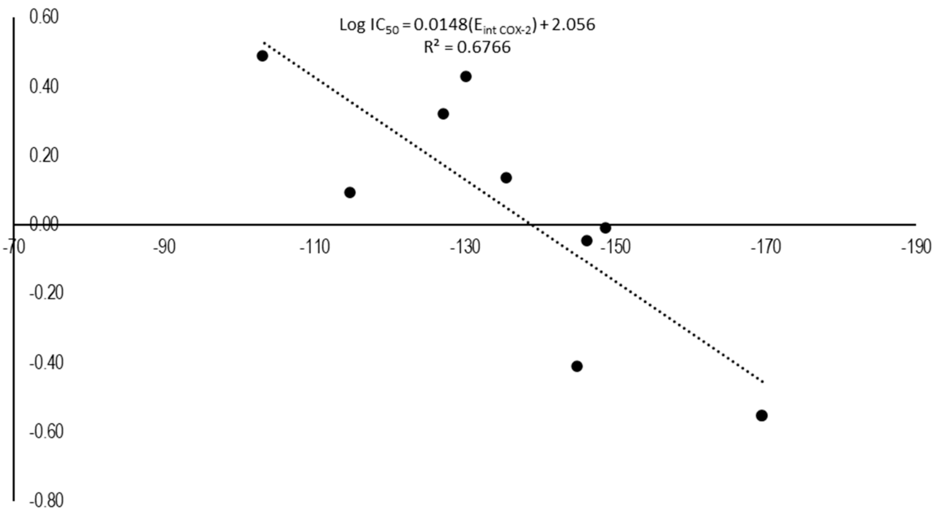

Figure S17.

Table S7. Interaction energy values (Kcal/mol) of COX-2 inhibitors that were coupled to the 3LN1 crystal. Log $IC_{50\,exp}$, Log IC_{50} calc and delta error (Δ error) values as show.

Compounds	Energy	Log $IC_{50\,exp}$	Log $IC_{50\,calc}$	Δ error
Celecoxib[1]	-169.55	-0.55	-0.45	0.1
16	-127.07	0.32	0.18	-0.14
20	-146.17	-0.05	-0.11	-0.06
21	-130.13	0.43	0.13	0.3
30	-148.72	-0.01	-0.15	-0.14
Diclofenac	-114.72	0.09	0.36	0.27
Flurbiprofen	-103.01	0.49	0.53	0.04
Meloxicam	-135.43	0.14	0.05	-0.09
Rofecoxib	-144.84	-0.41	-0.09	0.32

[1] Ligand corresponding to the crystal.

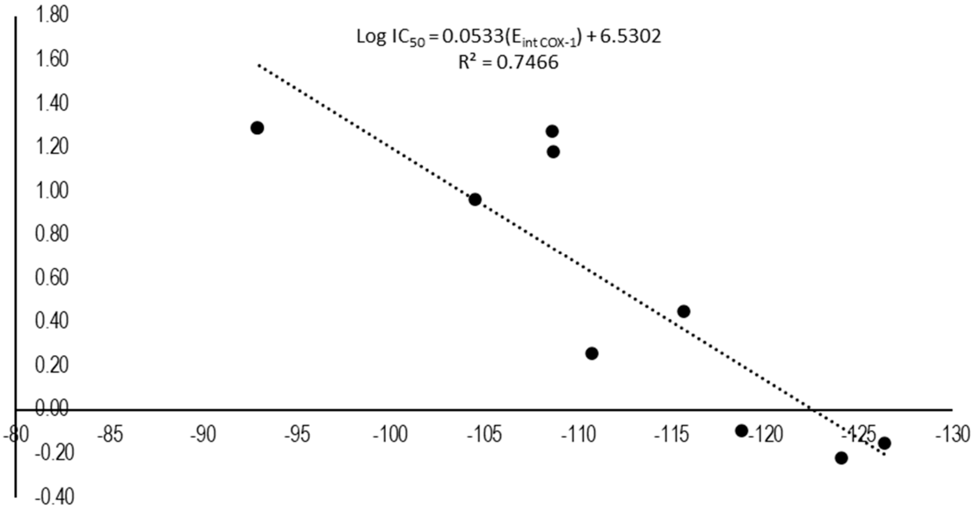

Figure S18.

Table S8. Interaction energy values (Kcal/mol) of COX-1 inhibitors that were coupled to the 4O1Z crystal. Log IC $_{50\,exp}$, Log IC$_{50\,calc}$ and delta error (Δerror) values as show.

Compounds	Energy	Log IC$_{50\,exp}$	Log IC$_{50\,calc}$	Δ error
Meloxicam[1]	-92.91	0.96	1.58	0.62
13	-110.750	0.26	0.63	0.37
16	-118.69	-0.10	0.20	0.3
19	-126.32	-0.15	-0.20	-0.05
20	-115.64	0.45	0.37	-0.08
21	-124.05	-0.22	-0.08	0.14
34	-108.68	1.18	0.74	-0.44
Diclofenac	-108.64	1.27	0.74	-0.53
Nimesulide	-104.48	0.96	0.96	0

[1] Ligand corresponding to the crystal.

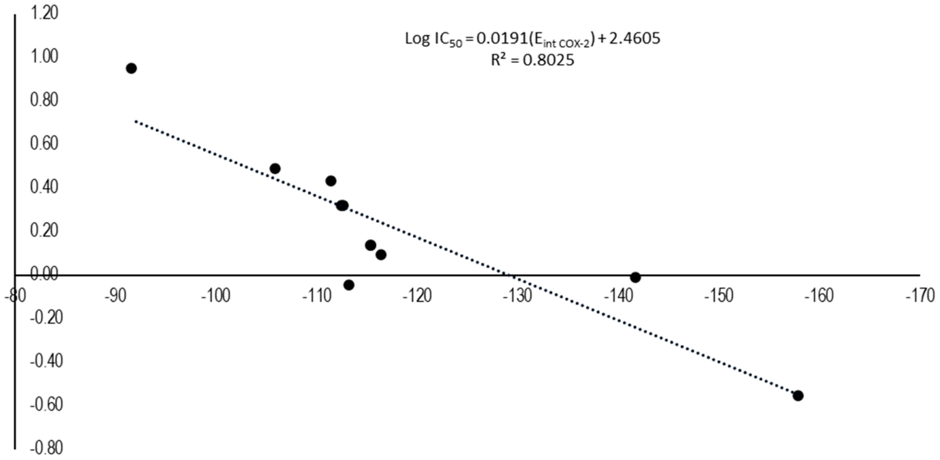

Figure S19.

Table S9. Interaction energy values (Kcal/mol) of COX-2 inhibitors that were coupled to the **4M11** crystal. Log IC$_{50\,exp}$, Log IC$_{50\,calc}$ and delta error (Δ error) values as show.

Compounds	Energy	Log IC$_{50\,exp}$	Log IC$_{50\,calc}$	Δ error
Meloxicam[1]	-115.40	0.14	0.26	0.12
13	-112.49	0.32	0.31	-0.01
16	-112.64	0.32	0.31	-0.01
20	-113.17	-0.05	0.30	0.35
21	-111.41	0.43	0.33	-0.1
30	-141.68	-0.01	-0.25	-0.24
Celecoxib	-157.82	-0.55	-0.55	0
Diclofenac	-116.44	0.09	0.24	0.15
Flurbiprofen	-105.91	0.49	0.44	-0.05
Ibuprofen	-91.59	0.95	0.71	-0.24

[1] Ligand corresponding to the crystal.

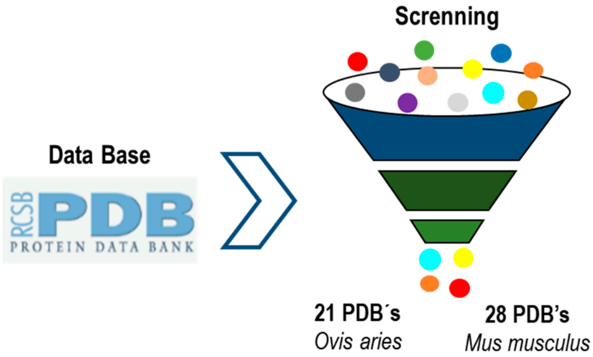

Figure S20. Search of crystals from COX-1 and COX-2.

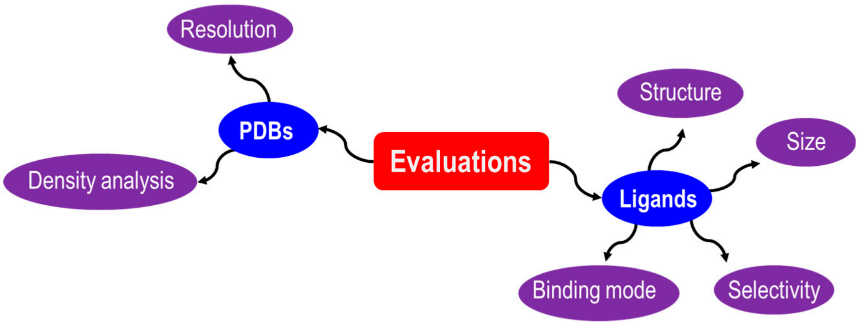

Figure S21. Analysis of crystals COXs with respect PDB and Ligands.

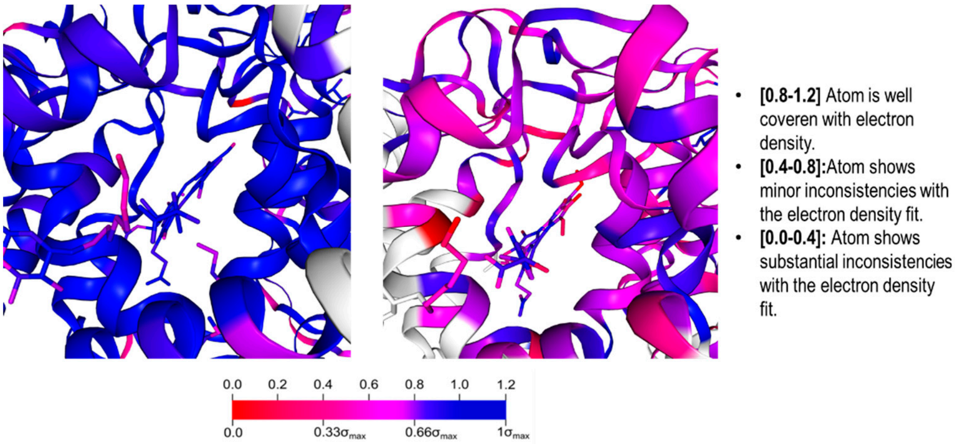

Figure S22. Examples of analysis about active site in crystals of COX-1 with EDIA. a) Ibuprofen (4PH9) with resolution of 2.6 Å, b) Flurbiprofen (3N8Z) with resolution of 2.9 Å.

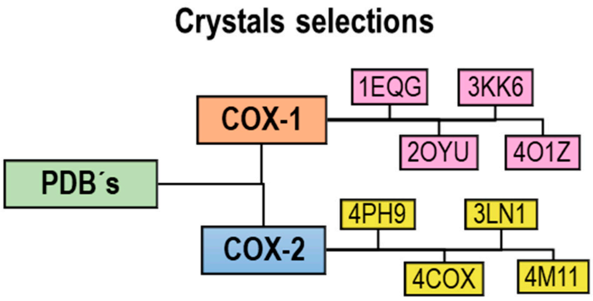

Figure S23. Final selection of crystals from computational model.

Table S10. Crystals and their flexible residues shown in the flexible docking.

PDBs	Flexible residues
**1EQG (COX-1)**	Phe381, Phe518, Ser353, Ser530, Trp387, Tyr355, Val116, Val349, Arg120, Ile523, Leu 352 Leu359, Leu531, Met522.
**4PH9 (COX-2)**	Phe519, Ser354, Ser531, Trp388, Tyr356, Tyr386, Val117, Val350, Val524, Arg121, Leu353, Leu360, Leu532, Met523.
**2OYU (COX-1)**	Leu531, Leu359, Leu352, Ile523, Ile517, Arg120, Val349, Tyr355, Gln192, Ser530, Ser516, Ser353.
**4COX (COX-2)**	Phe381, Phe518, Ser353, Ser530, Trp387, Tyr355, Tyr385, Val349, Val523, Arg120, Leu352, Leu384, Leu531, Met522.
**3KK6 (COX-1)**	His90, Phe381, Phe518, Ser353, Ser516, Ser530, Trp387, Gln192, Tyr355, Val116, Val349, Ile517, Ile523, Leu352, Leu359, Leu531, Met522.
**3LN1 (COX-2)**	His75, Phe367, Phe504, Ser339, Ser516, Trp373, Gln178, Tyr341, Tyr371, Val335, Val509, Arg499, Leu338, Leu345, Leu370, Leu517.
**4O1Z (COX-1)**	Ser353, Ser530, Trp387, Tyr355, Val116, Val349, Arg120, Ile345, Ile523, Leu117, Leu352, Leu359, Leu384, Leu531, Leu534, Leu535, Met113, Met522.
**4M11 (COX-2)**	Phe518, Ser530, Trp387, Tyr355, Val349, Val523, Arg120, Ile345, Leu117, Leu352, Leu359, Leu531, Leu534, Met113, Met522.
